# Cyclic heptapeptides with metal binding properties isolated from the fungus *Cadophora malorum* from Antarctic soil

**DOI:** 10.1007/s13659-022-00348-x

**Published:** 2022-07-14

**Authors:** Guidmar C. Donalle, María Martha Martorell, Gastón E. Siless, Lucas Ruberto, Gabriela M. Cabrera

**Affiliations:** 1 Facultad de Ciencias Exactas y Naturales, Departamento de Química Orgánica, Universidad de Buenos Aires, Buenos Aires, Argentina; 2 Unidad de Microanálisis y Métodos Físicos Aplicados a la Química Orgánica (UMYMFOR), CONICET-Universidad de Buenos Aires, Buenos Aires, Argentina; 3 Instituto Antártico Argentino, Instituto Nanobiotec, CONICET-Universidad de Buenos Aires, Buenos Aires, Argentina

**Keywords:** Cyclic peptide, *Cadophora malorum*, Metal binding

## Abstract

The Antarctic fungus *Cadophora malorum* produces previously undescribed cyclic heptapeptides (cadophorin **A** and **B**) containing an anthranilic acid residue. The planar structure of these peptides was determined by high-resolution mass spectrometry combined with extensive 1D and 2D NMR spectroscopy. The absolute configuration of the amino acids was determined by Marfey’s method, with HPLC analysis of FDVA (Nα-(2,4-dinitro-5-fluorphenyl)-l-valinamide) derivatives making use of a PFP column. Remarkably, cadophorin **2** possesses both the uncommon d-Ile and d-*allo*-Ile in its structure. The peptides have metal binding properties as shown by LCMS with *post column* addition of metal salt solutions. These results were supported by DFT calculations.

**Graphical Abstract:**

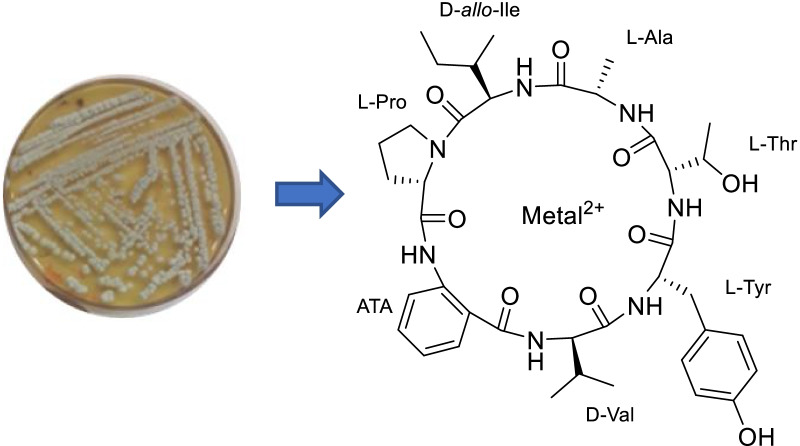

**Supplementary Information:**

The online version contains supplementary material available at 10.1007/s13659-022-00348-x.

## Introduction

Marine fungi are known to produce metabolites with a plethora of bioactivities, which include antimicrobial, antiviral, antitumoral, anti-inflammatory activities among others. Notably, marine natural products (MNP) from fungi represent nearly half of all reported MNP [1, 2]. Fungi isolated from Antarctic environments are not an exception and are also well-known as producers of bioactive metabolites [3, 4]. *Cadophora* is a worldwide ubiquitous genus with 43 species nowadays [5] some of which, including *C. malorum*, were reported from Antarctica locations [6]. Some *Cadophora* isolates have been reported to produce a series of bioactive metabolites with a wide range of structural scaffolds [7–9].

Metallomics integrates multidisciplinary research for the study of the relationships between bio-metals to bio-function. One of the fields of increasing interest in this area is the study of the metal coordination capability of metabolites, which may account for many biological activities such as metal hijacking, resistance to reactive oxygen species, production of sexual spores in microorganisms [10].

Electrospray ionization (ESI), a soft ionization technique able to transfer ions from solution to the gas phase, has been previously employed for the evaluation of the metal binding nature of secondary metabolites. Metal ions of alkali, alkaline earth and transition metals have been widely used in ESI, in order to record spectra of mixtures of metal salt solutions and samples, or by post-HPLC column addition of metal salt solutions [11, 12]. In particular, the latter technique has been applied as a screening method for the detection of compounds with metal binding properties [12, 13].

As part of the search for new natural products produced from Antarctica-derived fungi, the LC–MS runs of extracts obtained from a collection of 26 strains were chemometrically analysed employing MS-DIAL [14] and an outlier sample, *C. malorum*, was selected. Two new cyclic peptides (cadophorins A and B) were isolated and identified from this extract. Since there are previous reports of cyclic peptides with metal binding properties [15], cadophorins A and B were evaluated as potential metal binders of alkaline-earth metals, Zinc and Copper. The new peptides showed the capability to form complexes with all the metals, although the stability of the formed species was higher in the case of Copper, Zinc and Magnesium. Although metal binding properties may account for certain bioactivities like ionophoric action [16], metal binders can be useful as well as self-assembling structures to construct ion channels in supramolecular chemistry, and also as asymmetric catalysts [17, 18].

## Results and discussion

The organic extracts of small cultures of *C. malorum* and other 25 strains isolated from Antarctic soil samples were analysed by LCMS using Electrospray in positive and negative ion mode, and the runs were screened using the free platform MS-DIAL [14]. Principal component analysis (PCA), applied to the negative ion mode data, showed in the score plot an outlier strain, *C. malorum.* (Additional file 1: Fig. S1). A search on the ion table for unique metabolites, which were present in only one PCA class (Additional file 1: Fig. S2), and the PCA loading plot, exposed the presence of two metabolites of peptidic nature, according to their MS2 spectra, with molecular weights of 763 and 777.

**Fig. 1:**
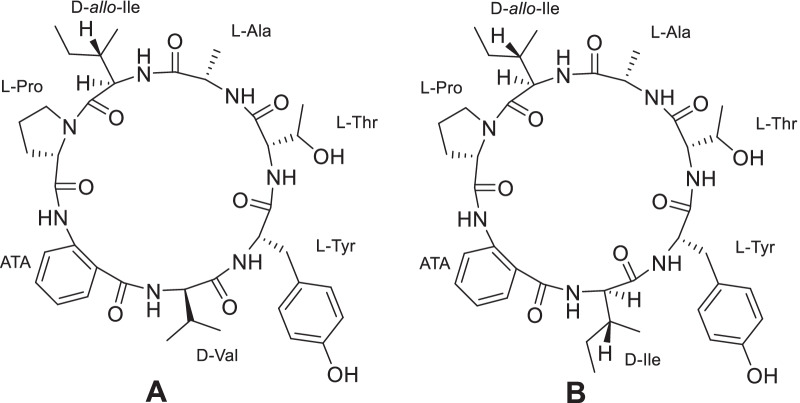
Cadophorins A and B isolated from *Cadophora malorum*. **A** is cadophorin A and **B** is cadophorin B

Based on this result, the components of the organic extract of *C. malorum* were separated by HPLC yielding two compounds, which were named cadophorins A and B (Fig. 1). Both compounds were considered pure after analysis by LCMS and ^1^H NMR in CDCl_3_/CD_3_OD. However, some duplicated nonexchangeable signals were observed in the ^1^H NMR spectra in DMSO-*d*_*6*_ or CD_3_OD (Additional file 1: Fig. S3.8). This fact indicated that the compounds exist in a slow conformational equilibrium in these solvents [19].

**Fig. 2:**
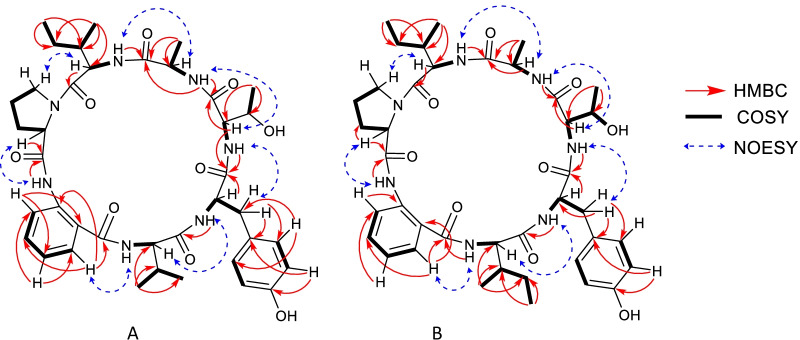
Relevant correlations observed in 2D NMR experiments. **A** is cadophorin A and **B** is cadophorin B

Cadophorin A (cadA) has a molecular formula of C_39_H_54_N_7_O_9_ as determined by HRESIMS. The MS/MS spectrum showed the presence of characteristic signals corresponding to the loss of some amino acids like alanine (Ala), threonine (Thr), tyrosine (Tyr), valine (Val) and leucine or isoleucine (Ile), although the structure could not be fully determined considering proteinogenic amino acids only. The ^13^C NMR (Table 1) and HSQC-DEPT spectra exhibited 12 aromatic carbon signals accounting for two phenyl groups, seven carbonyl signals and five methylene, nine methine and six methyl group signals (Table 1). The 2D NMR data (COSY, HSQC and HMBC) established substructures matching with Ala, Thr, Tyr, Val, Ile, Pro and an anthranilic acid residue (ATA). This last fragment exhibited a characteristic system of four consecutive aromatic protons (*δ*_H_ 8.48, br d, *J* = 8.0 Hz; *δ*_H_ 7.49, dt, *J* = 8.0, 1.3 Hz; *δ*_H_ 7.13, dt, *J* = 8.0, 1.0 Hz; *δ*_H_ 7.79, dd, *J* = 8.0, 1.3 Hz). The sequence of the amino acids was established by analyses of HMBC and NOESY correlations, in combination with Mass Spectrometry. An Ala was positioned between Thr and Ile by the HMBC correlations of Ala-NH (*δ*_H_ 7.42 ppm) to C-1 (*δ*_C_ 170.3) of Thr and of Ile-NH (*δ*_H_ 8.23 ppm) to C-1 of Ala (*δ*_C_ 172.0 ppm). In the same way, Thr was attached to a Tyr by the HMBC correlations of Thr-NH (*δ*_H_ 8.33 ppm) and Thr-H-2 (*δ*_H_ 4.05 ppm) to Tyr-C-1 (*δ*_C_ 171.9). At the same time, Tyr-NH (*δ*_H_ 8.18 ppm) correlated to Val-C-1 (*δ*_C_ 170.2). A NOESY correlation of Val-NH (*δ*_H_ 8.13 ppm) to ATA-H-3 (*δ*_H_ 7.79 ppm) allowed us to place Val next to ATA. Moreover, the connectivity between ATA and Pro was identified based on an HMBC correlation of ATA-NH (12.03 ppm) and Pro-C-1 (*δ*_C_ 170.6 ppm). Finally, a NOESY correlation between Pro-H-5 (*δ*_H_ 3.67–3.69 ppm) and Ile-H-2 (*δ*_H_ 1.85 ppm) connected Pro to Ile and closed the cycle. Other HMBC and NOESY correlations supported this planar structure (Fig. 2 and Additional file 1: Figs. S3.1–S3.7, S3.9–S3.11), as well as a detailed analysis of the MS/MS spectrum (Additional file 1: Table S3.1). The *trans* configuration of Pro can be deduced from the difference between *δ*_C_ C-3 and *δ*_C_ C-4 [15].

**Table 1:** ^13^C NMR (500 MHz) and ^1^H NMR (125 MHz) data for cadophorins A and B

	Number	Cadophorin A		Cadophorin B	
		*δ*_C_, type	*δ*_H_, (*J* (Hz))	*δ*_C_, type	*δ*_H_, (*J* (Hz))
l-Ala	NH		7.42, d (6.5)		7.42, d (6.5)
	1	172.0, CO		172.0, CO	
	2	49.2, CH	4.32, qi (6.5)	49.2, CH	4.32, qi (6.5)
	3	20.9, CH_3_	1.32, d (6.5)	20.8, CH_3_	1.32, d (6.5)
d-*allo*-Ile	NH		8.23, d (9.4)		8.22, d (9.5)
	1	170.7, CO		170.7, CO	
	2	52.3, CH	5.27, dd (9.4, 2.6)	52.3, CH	5.27, dd (9.5, 2.4)
	3	37.3, CH	1.85, m	37.3, CH	1.85, m
	4	27.0, CH_2_	1.21, m	27.0, CH_2_	1.21, m
	5	12.4, CH_3_	1.03, t (7.1)	12.3, CH_3_	1.03, t (7.2)
	6	15.1, CH_3_	0.83, d (6.9)	15.1, CH_3_	0.83, d (6.8)
l-Pro	1	170.6, CO		170.6, CO	
	2	62.2, CH	4.40, dd (8.6, 1.5)	62.2, CH	4.40, dd (8.7, 1.7)
	3	29.8, CH_2_	2.12, 2.01, m	29.9, CH_2_	2.12, 2.01, m
	4	24.1, CH_2_	1.97, 1.76, m	24.1, CH_2_	1.97, 1.75, m
	5	46.6, CH_2_	3.71, 3.67 m	46.7, CH_2_	3.72, 3.67, m
ATA	NH		12.03, s		11.99, s
	1	168.3, CO		168.3, CO	
	2	119.7, C		119.7, C	
	3	129.2, CH	7.79, dd (8.0, 1.3)	129.2, CH	7.76, dd (8.0, 1.3)
	4	123.0, CH	7.13, dt (8.0, 1.0)	123.0, CH	7.12, dt (8.0, 1.1)
	5	132.9, CH	7.49, dt (8.0, 1.3)	132.9, CH	7.48, dt (8.0, 1.1)
	6	119.3, CH	8.48, *br* d (8.0)	119.3, CH	8.48, d (8.0)
	7	139.5, C		139.5, C	
d-Val	NH		8.13, d (9.0)		
	1	170.2, CO			
	2	57.0, CH	4.78, dd (9.0, 4.0)		
	3	31.8, CH	2.08, m		
	4	19.9, CH_3_	0.92, d (6.8)		
	5	17.0, CH_3_	0.50, d (6.8)		
d-Ile	NH				8.15, d (8.9)
	1			170.2, CO	
	2			57.0, CH	4.77, dd (8.9, 4.4)
	3			38.4, CH	1.8, m
	4			23.7, CH_2_	0.93, m
	5			12.0, CH_3_	0.74, t (7.2)
	6			15.8, CH_3_	0.86, d (6.7)
l-Tyr	NH		8.16, d (10.2)		8.16, d (10.1)
	1	171.9, CO		171.9, CO	
	2	56.8, CH	4.62, dt (10.2, 5.2)	56.8, CH	4.65, dt (10.1, 5.2)
	3	36.5, CH_2_	2.96, 2.93, m_AB_	36.5, CH_2_	2.95, m
	4	128.1, C		128.0, C	
	5	130.3, CH	7.06, d (8.5)	130.3, CH	7.04, d (8.6)
	6	115.5, CH	6.66, d (8.5)	115.4, CH	6.66, d (8.6)
	7	156.4, C		156.4, C	
l-Thr	NH		8.33, d (9.6)		8.37, d (9.6)
	1	170.3, CO		170.3, CO	
	2	59.0, CH	4.05, dd (9.6, 2.4)	59.0, CH	4.06, dd (9.6, 2.3)
	3	66.8, CH	4.12, m	66.8, CH	4.12, m
	4	21.2, CH_3_	1.02, d (6.0)	21.2, CH_3_	1.02, d (6.1)
	OH		5.15, d (4.3)		5.18, s

The absolute configuration of each amino acid was determined via Marfey’s analysis. Cadophorin A was hydrolysed, and the hydrolysate was derivatized with *N*-(2,4-dinitro-5-fluorophenyl)-l-valinamide (l-FDVA, Marfey’s reagent) followed by HPLC–DAD/MS analysis and comparison with authentic standards of Marfey’s derivatized amino acid [20]. For the separation of the diasteromeric d-*allo*-Ile and d-Ile MR derivatives, a PFP HPLC column was employed [21]. This experiment revealed the presence of l-Ala, l-Thr, l-Tyr, d-Val, l-Pro and d-*allo*-Ile in cadophorin A (Table 2). The presence of d-*allo*-Ile was confirmed by co-injection of the l-FDVA derivative of d-*allo*-Ile and cadophorin A. Although PFP phase has been used previously for this application [21], in this work a different set of chromatographic conditions allowed a better differentiation of the four stereoisomers of isoleucine. Temperature is also an important factor for this separation, and higher temperatures were counterproductive in this respect.

**Table 2:** Table retention times (R_T_) of amino acid l-FDVA derivatives

Amino acid	Theoretical m/z [M+H]^+^	Molecular formula [M+H]^+^	Standard amino acid		Cadophorin A			Cadophorin B		
			Serie L	Serie D	m/z	T_R(min)_	Error (ppm)	m/z	T_R(min)_	Error (ppm)
			T_R(min)_	T_R(min)_						
Ala	370.1357	C_14_H_19_N_5_O_7_	13.3	16.5	370.1349	13.4	2.3	370.1343	13.5	3.9
Pro	396.1513	C_16_H_21_N_5_O_7_	12.6	15.1	396.1532	12.6	4.5	396.1505	12.6	2.3
*allo*-Ile	412.1826	C_17_H_25_N_5_O_7_	19.2	23.4	412.1807	23.5	4.7	412.1810	23.4	4.1
Ile	412.1826	C_17_H_25_N_5_O_7_	19.4	23.6	–	–	–	412.1825	23.6	0.5
Thr	400.1462	C_15_H_21_N_5_O_8_	8.4	12.9	400.1474	8.4	2.9	400.1466	8.4	0.9
Tyr*	742.2427	C_31_H_35_N_9_O_13_	29.5	32.7	742.2408	29.5	2.5	742.2430	29.5	0.5
Val	398.1670	C_16_H_23_N_5_O_7_	17.0	21.5	398.1647	21.0	6.0	–	–	–

^*^Disubstituted derivative

Cadophorin B (cadB) showed a signal m/z 778.4135 corresponding to a protonated molecule with a molecular formula C_40_H_55_N_7_O_9_ in the ESI-HR spectrum. The NMR spectra of cadophorin B were very similar to those of cadophorin A. The main difference was the absence of the methyl signals of d-Val and the appearance of the signals of two methyl groups at *δ*_H_ 0.86 (d) and 0.74 (t) in the ^1^H NMR spectrum (Table 1), inferring the presence of an Ile instead of Val. 2D NMR spectra and MS and MS2 data confirmed this assumption (Additional file 1: Figs S4.1–S4.12) and all the correlations in the HMBC and NOESY spectra also corroborated the similarity between cadA and cadB (Fig. 2). Cadophorin B was hydrolised and derivatised in the same way as cadA and the analysis of the l-FDVA derivatised amino acids revealed a similar absolute configuration of the amino acids as in cadA and the presence of an additional d-Ile. Co-injection with authentic samples of derivatised d-Ile and d-*allo*-Ile showed that both aminoacids were present (Additional file 1: Fig. S5). Considering the similarity of cadophorin A and B, and the way this kind of peptides are biosynthesed [22], it can be assumed that D-Ile is in the same position as d-Val in cadophorin A.

The presence of d-*allo*-Ile in cyclic peptides produced by microorganisms is quite common, however this is not the case for d-Ile. The monamycins, which are antibiotic hexapeptides are probably the only example of natural cyclic peptides incorporating this amino acid [23]. d-Ile has an opposite configuration at C-3 compared to l-Ile or d-*allo*-Ile. Since this position is not vicinal to the carboxyl group of the aminoacid, d-Ile cannot be biosynthetised by the typical enolisation/epimerization sequence. Instead, biosynthetic studies have shown that the epimerization at C-3 occurs through an α-keto acid intermediate [24]. Since the report of the monamycins, other cyclic peptides containing this amino acid were informed, although their structures were not adequately confirmed or were incorrect [25, 26].

The cyclization of a linear peptide normally contributes to substantial conformational rigidity over the linear form, which explains the specificity of cyclic peptides for target sites and their increased resistance towards proteases. Proline itself gives a strong conformational rigidity compared to other amino acids, but the presence of ATA attached to Pro in a cyclic peptide further restricts the flexibility of the molecules. 3D structures of the lower energy conformers of cad1 are shown in Additional file 1: Fig. S7.1.

There are a few examples of cyclic peptides with this sequence ATA-Pro or p-hydroxy ATA-Pro, like the tricyclic peptide psychrophilin A, isolated from the psychrotolerant fungus *Penicillium ribeum* [27] and asperpetide A from a gorgonian-derived *Aspergillus* sp. [28].

Cadophorins were analysed by LCMS with post-column *in source* addition of metal chloride solutions of Magnesium, Calcium, Strontium, Copper, and Zinc to test their metal binding properties. These metals, except for Strontium, were selected due to their metabolic importance in living organisms, while Strontium was included in order to compare its performance with the other alkaline earth metals. The mass spectra showed the presence of a signal corresponding to the ion [cad + Me]^2+^, where Me is any metal, in all the cases as the main signal (Additional file 1: Fig. S6.1). The response of that signal was plotted for cadB giving a descending order of response from Sr, Ca, Mg, Zn to Cu (Additional file 1: Fig. S6.2). This response is related to the ease of ionization. When comparing related compounds, the less polar and less solvated species are nearest to the surface of the electrosprayed droplet and are easily ionised. In this sense, the relationship between the responses of alkaline earth complexes could be predicted (Sr > Ca > Mg). These MS experiments are also strongly influenced by the kinetics, as the complexes are formed in situ without time for equilibration, and for this reason the comparison between Mg, Cu and Zn complexes is not straightforward and would require additional kinetic studies.

**Fig. 3:**
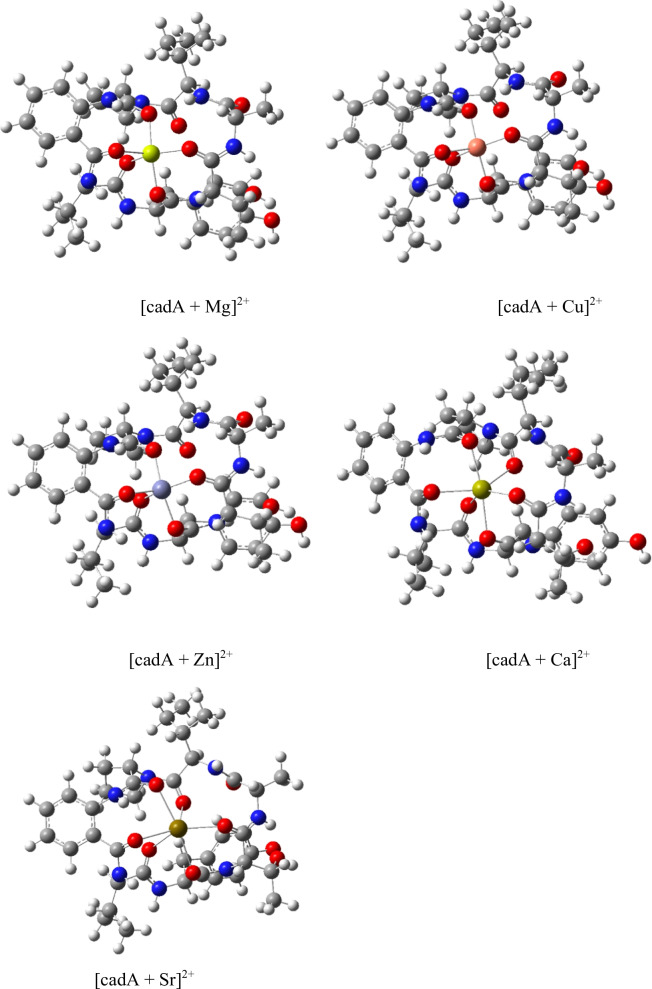
Optimised structures of cadA–Me complexes calculated at the B3LYP level of theory, Me = Mg^2+^ and Cu^2+^

The optimised structures of the complexes of cadA with different metals were calculated by DFT methods to correlate the MS results with the stability of the complexes, which are shown in Fig. 3. The site of coordination to the divalent cation was the oxygen of carbonyl moieties in all the cases. The geometry observed around the metal center was trigonal bipyramidal for Mg, Cu and Zn, with carbonyls of ATA, Val, Thr in equatorial and Pro and Tyr in the axial positions. In the case of Ca and Sr, an additional coordination to the carbonyl of the *allo*-Ile resulted in a distorted octahedral geometry. The relevant interatomic distances for the metal complexes are shown in Additional file 1: Table S7.1. These geometries and the distances from the cation to the carbonyl oxygen are typical for these metal cations [29].

For most of the complexes [cadA + Me]^2+^ (Me = Mg, Ca, Sr, Zn), an additional isomer of higher energy was found, also with trigonal bipyramidal geometry, which coordinates the divalent cation with the carbonyls of Pro, Ser and Val equatorially and Tyr and *allo*-Ile in the axial positions. This additional mode of coordination holds the metal in a central position, which is less exposed to the surface of the molecule (Additional file 1: Table S7.2).

The calculated energies for the lower energy conformers are shown in Table 3, and show an order of stabilities: Cu > Zn > Mg > Ca > Sr. These results indicate that the metal complexes of cadophorins are more stable in the case of the smaller metal cations Cu, Zn and Mg which have similar ionic radii.

**Table 3:** Optimised energies for cadA–Me complexes [cadA + Me]^2+^ calculated at the B3LYP level of theory, Me = Mg^2+^, Ca^2+^, Sr^2+^, Zn^2+^ and Cu^2+^ for: cadA + Me(H_2_O)_6_^2+^ →  [cadA + Me]^2+^ + 6 H_2_O

Me	Base	*ΔG°* (kcal/mol)	*ΔH°* (kcal/mol)
Mg	6-31G(d)	− 40.21	− 1.17
Ca	6-31G(d)	− 37.78	− 2.76
Sr	LANL2DZ	− 20.38	14.51
Sr	6-31G(d)/LANL2DZ	− 8.05	24.84
Zn	6-31G(d)	− 47.72	− 12.15
Cu	6-31G(d)	− 59.51	− 20.60

It is worth to mention that the employed MS methodology allowed the evaluation of the binding properties of cyclic peptides present in small amounts, with the advantage of allowing hundreds of experiments using the same sample.

Cadophorins A and B showed antifungal activity against *Candida albicans* and *C. haemulonii* at 20 µg/spot (Additional file 1). The extract of *C. malorum* possessed also antifungal activity against phytopathogenic strains, which was attributed to the presence of wortmannin, a known antifungal metabolite, also present in the extract (Additional file 1: Fig. S8) [30].

## Experimental section

### General experimental procedures

Optical rotations were recorded on a PerkinElmer 343 polarimeter. Electronic Circular Dichroism spectra were determined in a Jasco J815 Spectropolarimeter. ^1^H- and ^13^C-NMR spectra were obtained on a Bruker Avance Neo 500 spectrometer operating at 500 MHz and 125 MHz, respectively; chemical shifts (δ_H_ and δ_C_) are informed in ppm, *J* in Hz. Two-dimensional NMR spectra (COSY, HSQC-DEPT, NOESY and HMBC) were recorded using standard Bruker software. HR (ESI) mass spectra and HPLC–MS runs were recorded using a MicrOTOF QII Bruker mass spectrometer. All solvents were distilled before use. LC/MS-grade methanol and water were purchased from Carlo Erba (Milan, Italy). Formic acid (p.a., ACS) was purchased from Merck (Merck KGaA, Darmstadt, Germany). Standard amino acids were purchased from Sigma-Aldrich (Merck KGaA, Darmstadt, Germany), except for d-Isoleucine which was purchased from TCI (Tokyo Chemical Industry Co., Ltd, Alpharetta, USA).

### Isolation and cultivation of Antarctic fungi

Soil samples were collected at Potter Peninsula, 25 de Mayo/King George Island (62° 14′ 18″ S, 58° 40′ 00″ W) Antarctica by M.M.M. and L.R.. For experimental details, see Additional file 1. The strain of *C. malorum* was classified by M.M.M. and L.R., deposited in the Culture Collection at the Argentinean Antarctic Institute (IAA) and the DNA sequences were submitted to GenBank under Accession Number OM177061.

### Cultivation of *Cadophora malorum*

For the large-scale cultures used for compound isolation, *Cadophora malorum* was grown on PDA for at 15 °C for 14 days. From these plates, small plugs of the culture were transferred into Erlenmeyer flasks containing Potato Dextrose Broth (PDB) and incubated at 15 °C and 200 rpm for 4 weeks. Supernatant was separated from the biomass by centrifugation at 4000 rpm for 10 min.

### Extraction and isolation of the metabolites.

The supernatant (1.5 L) was extracted with ethyl acetate (3 × 500 mL) and the organic extract (100 mg) was subjected to HPLC (column: YMC C18, 5 µm, 22.5 × 2.5 cm; MeOH-H_2_O 55:45) yielding cadophorin B (1.8 mg), cadophorin A, re-purified by the same technique (1.0 mg), and a fraction which was separated by prep. TLC (MeOH:CH_2_Cl_2_ 5:95) yielding 2.4 mg of wortmannin.

### Marfey’s derivatives

A sample of cadA and cadB (approximately 0.5 mg) were dissolved in 2.0 mL of 6 M hydrochloric acid and hydrolyzed overnight at 110 °C. The hydrolysates were treated with Marfey’s reagent (FDNP-Val-NH_2_) as previously reported [31]. The derivatives were analysed by LC–DAD–MS (column: Luna PFP, 3 μm, 2.0 mm × 100 mm; Phenomenex, Torrance, CA, USA), set at 28 °C. The mobile phase was H_2_O containing 0.1% formic acid (A) and ACN (B), at a flow rate of 0.3 mL/min. A linear gradient elution was performed as follows: 25% B (3 min), 25–65% B (3–40 min), 100% B (41–45 min), 25% B (45–50 min).

The same procedure was repeated for cadophorin B (0.2 mg) to confirm the results.

### Binding metal experiments

The MS instrument with an ESI source was operated using the following conditions: capillary voltage 4.5 kV; end plate offset 500 V; dry temperature 200 °C. Nitrogen was used as dry gas (11.0 L/min) and as nebuliser gas (nebuliser pressure 3.4 bar). Data acquisition and processing were performed using Bruker Compass Data Analysis software. A Luna PFP HPLC column (3 μm, 2.0 mm × 100 mm; Phenomenex, Torrance, CA, USA), set at 30 °C, was employed as the stationary phase. The mobile phase was water containing 0.1% formic acid (A) and MeOH (B), at a flow rate of 0.3 mL/min. Linear gradient elution was performed as follows: 55% B (0–2 min), 55–100% (2–7 min), 100% (7–14 min).

Post-column addition of metal solutions. An aqueous solution (10 mM) of MgCl_2_, CaCl_2_, SrCl_2_, CuCl_2_ or ZnCl_2_ was introduced using a syringe pump at a flow rate of 3 μL/min, via a T-junction before entrance into the ion source. The solutions of metals were previously titrated with an EDTA solution employing murexide (Ca^2+^ and Cu^2+^) or Eriochrome Black T (Mg^2+^, Zn^2+^) as indicators.

### Antifungal assays

For experimental details, see Additional file 1.

### Computational section

See Additional file 1.

### Compound information

*Cadophorin* A. White amorphous solid; ${[\alpha]_{\text{D}}^{20}}$ − 11.8 (*c* 0.065, CHCl_3_). UV (MeOH) λ_max_ (log ε) 271 (3.0), 245 (3.3) nm. ^1^H NMR and ^13^C NMR (see Table 1). Positive ion HRESIMS m/z 764.3983 (Calcd for C_39_H_54_N_7_O_9_ [M+H]^+^, 764.3978).

*Cadophorin* B. White amorphous solid; ${[\alpha]_{\text{D}}^{20}}$ − 35.1 (*c* 0.080, CHCl_3_). UV (MeOH) λ_max_ (log ε) 271 (3.0), 245 (3.4) nm. ^1^H NMR and ^13^C NMR (see Table 1). Positive ion HRESIMS m/z 778.4134 (Calcd for C_40_H_56_N_7_O_9_ [M+H]^+^, 778.4108).

## Supplementary Information

**Additional file 1. **PCA analysis (score plot); Spectroscopical data (^1^H NMR, ^13^C NMR, ^1^H,^1^H COSY, HSQC, HMBC, NOESY, HRMS (ESI), MS/MS spectra, ECD) of cadophorins A and B; Comparison of the retention times of the L-FDVA derivatives of d-Ile, d-*allo*-Ile present in cadophorin B, to co-injected standards; MS spectra of cadophorin B with post-column *in source* addition of metal salt solutions; Optimized structures and geometrical parameters of metal complexes calculated at the B3LYP level of theory; ^1^H NMR and ^13^C NMR of wortmannin; Isolation and cultivation of Antarctic fungal strains; Antifungal assay.
