# Supplementary material for: Cyclic heptapeptides with metal binding properties isolated from the fungus Cadophora malorum from Antarctic soil
Source: Nat Prod Bioprospect. 2022 Jul 14;12(1):26. doi: 10.1007/s13659-022-00348-x (PMC9279517; doi:10.1007/s13659-022-00348-x)
Supplement: Supplementary file 1 — Additional file 1. PCA analysis (score plot); Spectroscopical data (1H NMR, 13C NMR, 1H,1H COSY, HSQC, HMBC, NOESY, HRMS (ESI), MS/MS spectra, ECD) of cadophorins A and B; Comparison of the retention times of the L-FDVA derivatives of d-Ile, d-allo-Ile present in cadophorin B, to co-injected standards; MS spectra of cadophorin B with post-column in source addition of metal salt solutions; Optimized structures and geometrical parameters of metal complexes calculated at the B3LYP level of theory; 1H NMR and 13C NMR of wortmannin; Isolation and cultivation of Antarctic fungal strains; Antifungal assay. [file 13659_2022_348_MOESM1_ESM.pdf]

Cyclic heptapeptides with metal binding properties from the fungus *Cadophora malorum* isolated from Antarctic soil

Guidmar C. Donalle[a,b], María Martha Martorell[c,d], Gastón E. Siless[a,b], Lucas Ruberto[c,d], Gabriela M. Cabrera[a,b]

[a] Facultad de Ciencias Exactas y Naturales, Departamento de Química Orgánica, Universidad de Buenos Aires, Buenos Aires, Argentina; [b] CONICET-Universidad de Buenos Aires, Unidad de Microanálisis y Métodos Físicos aplicados a la Química Orgánica (UMYMFOR), Buenos Aires, Argentina; [c] Instituto Antártico Argentino; [d] Instituto Nanobiotec (UBA-CONICET).

Key words: cyclic peptide, *Cadophora malorum*, Metal binding

Supplementary material

Part 1. Figure 1S. Principal component analysis (Score plot) of a collection of Antarctic fungal strains.

Part 2. Figure 2S. Presence of the signal m/z 762 (cadophorin A) in the extract of *C. malorum* (CM) at 25.8 min compared with the other extracts of the Antarctic collection.

Part 3. Spectroscopic data (^{1}H NMR, ^{13}C NMR, COSY, HSQC, HMBC, NOESY, HR-ESI-MS, MS2 m/z 764, ECD) of cadophorin A

Part 4. Spectroscopic data (^{1}H NMR, ^{13}C NMR, COSY, HSQC, HMBC, NOESY, HR-ESI-MS, MS2 m/z 778) of cadophorin B

Part 5. Comparison of the retention times of the L-FDVA derivatives of D-Ile, D-*allo*-Ile present in cad2 to co-injected standards of L-FDVA derivatives of D-Ile and D-*allo*-Ile.

Part 6. MS spectra of cadB with post-column *in source* addition of metal salt solutions

Part 7. Computational methods. Optimized structures of cadA and geometrical parameters of cadA-Me complexes calculated at the B3LYP level of theory, Me= Mg^{2+}, Ca^{2+}, Sr^{2+}, Zn^{2+} and Cu^{2+}

Part 8. ^{1}H NMR and ^{13}C NMR of wortmannin

Part 9. Isolation and cultivation of Antarctic fungal strains

Part 10. Antifungal assay

Part 1. Figure 1S. Principal component analysis (Score plot) of a collection of Antarctica fungal strains.

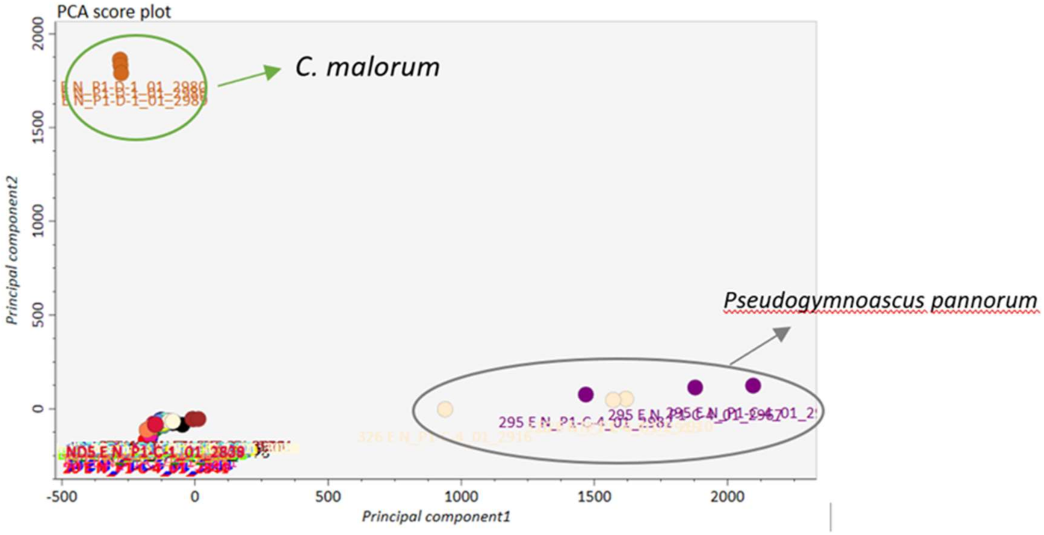

MS DIAL (Tsugawa Hiroshi, Cajka Tomas, Kind Tobias, Ma Yan, Higgins Brendan, Ikeda Kazutaka, Kanazawa Mitsuhiro, Vander Gheynst Jean, Fiehn Oliver, Arita Masanori. MS-DIAL: data independent MS/MS deconvolution for comprehensive metabolome analysis. Nature Methods, 12, 523–526, 2015) was employed to obtain these data.

The MS instrument (Experimental Section) was operated using the following conditions: capillary voltage 3.5 kV; end plate offset -500 V; dry temperature 200°C. Nitrogen was used as dry gas (11.0 L/min) and as nebuliser gas (nebuliser pressure 3.4 bar). Data acquisition and processing were performed using Bruker Compass Data Analysis software. A Luna C18 HPLC column (3 μm, 2.0 mm × 100 mm; Phenomenex, Torrance, CA, USA), set at 30°C, was employed as the stationary phase. The mobile phase was H_2O containing 0.1% formic acid (A) and MeOH (B), at a flow rate of 0.3 mL/min. Linear gradient elution was performed as follows: 10% B (0-2 min), 10-75% B (2-25 min), 100% B (26-44 min), 10% B (45-50 min).

Part 2. Figure 2S. Signal m/z 762 (cadophorin A) in the extract of *C. malorum* (CM) at 25.8 min compared with the other extracts of the Antarctic collection.

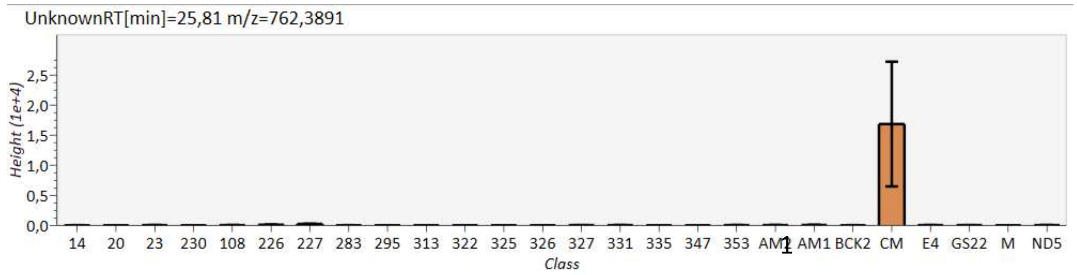

Part 3. Spectroscopic data of cadophorin A

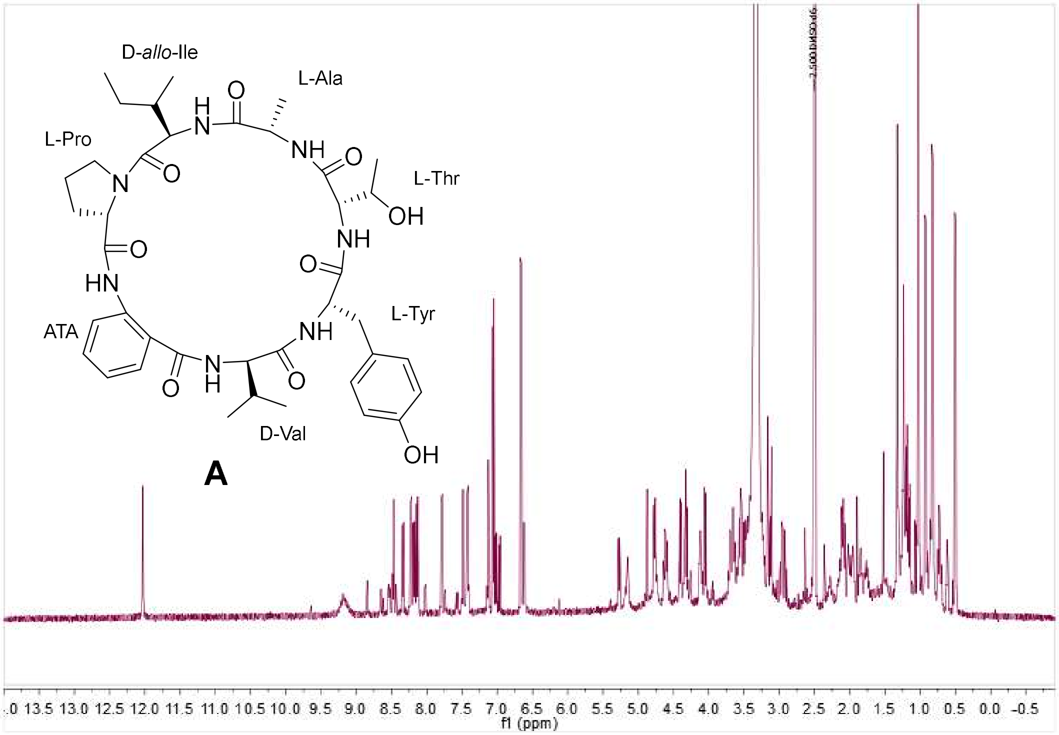

Figure S3.1. ^{1}H NMR spectrum of cadophorin A (DMSO-d_6)

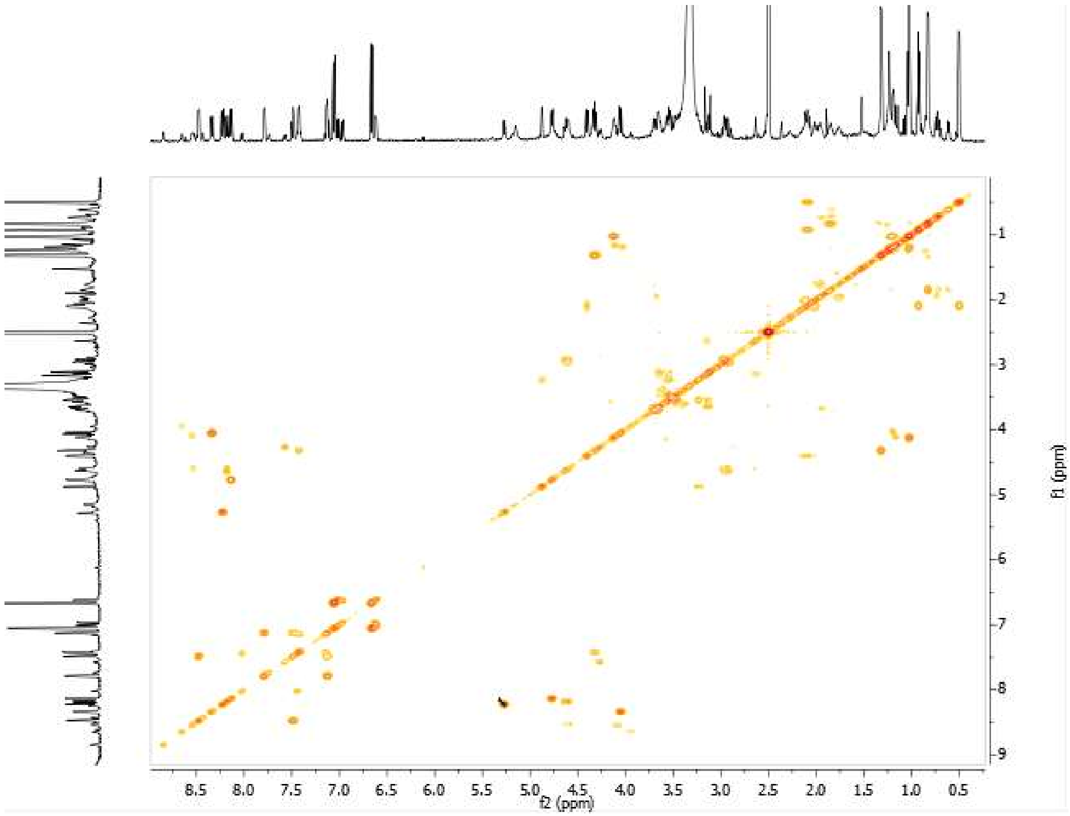

Figure S3.2. COSY spectrum of cadophorin A

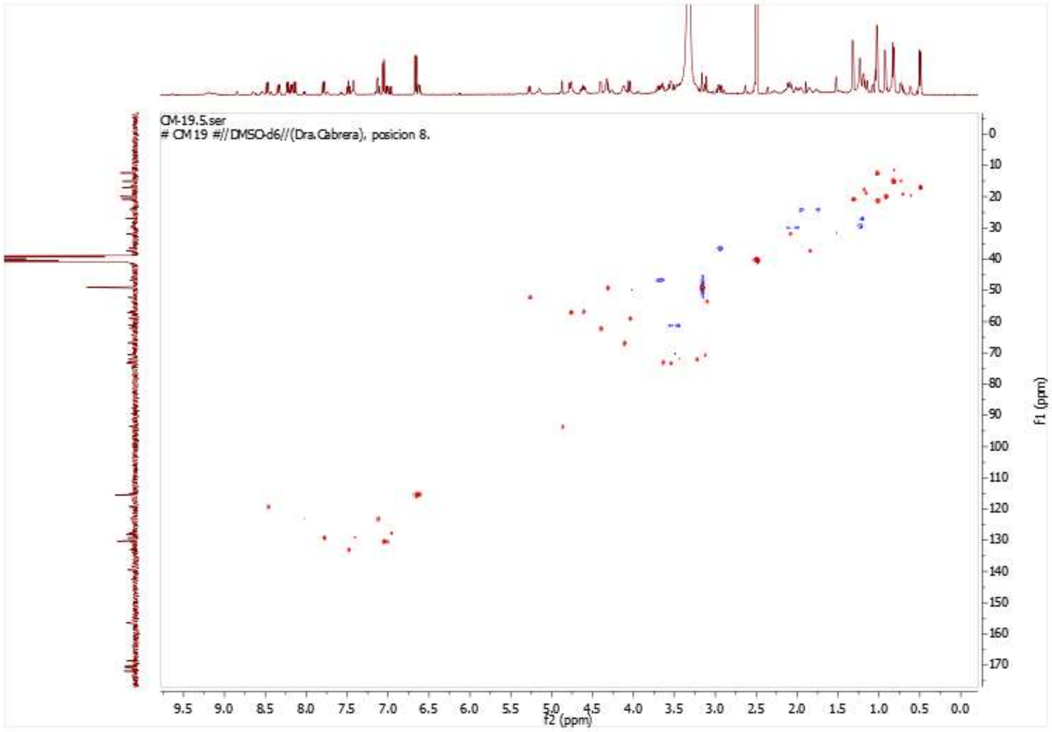

Figure S3.3. HSQC-DEPT spectrum of cadophorin A

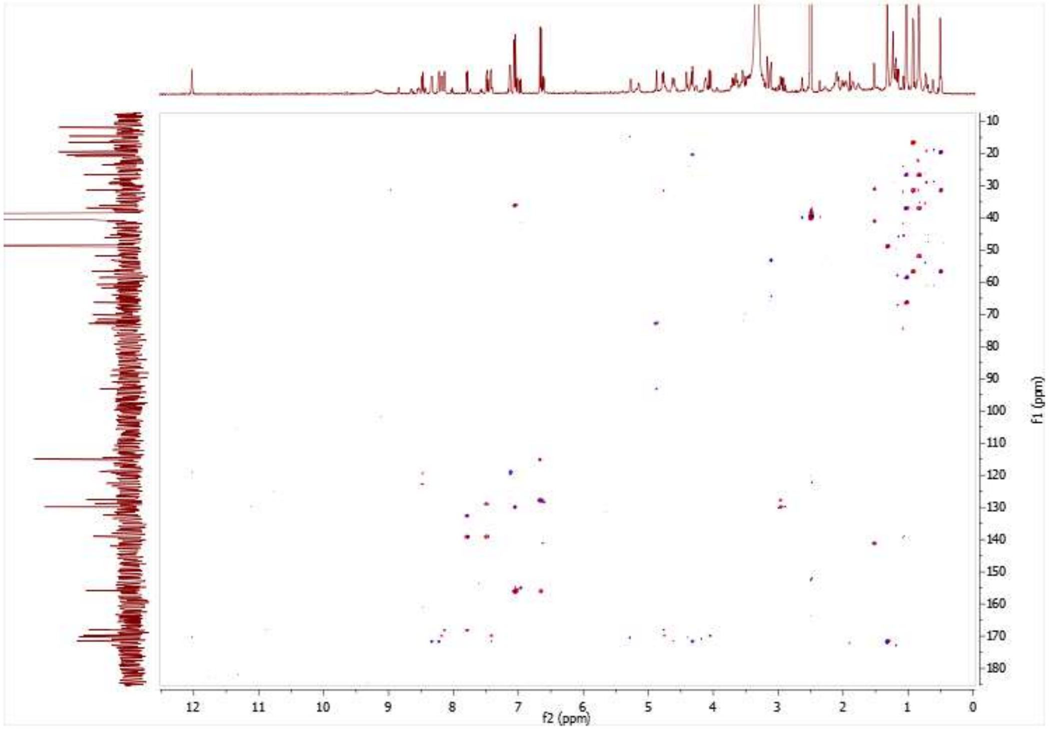

Figure S3.4. HMBC spectrum of cadophorin A

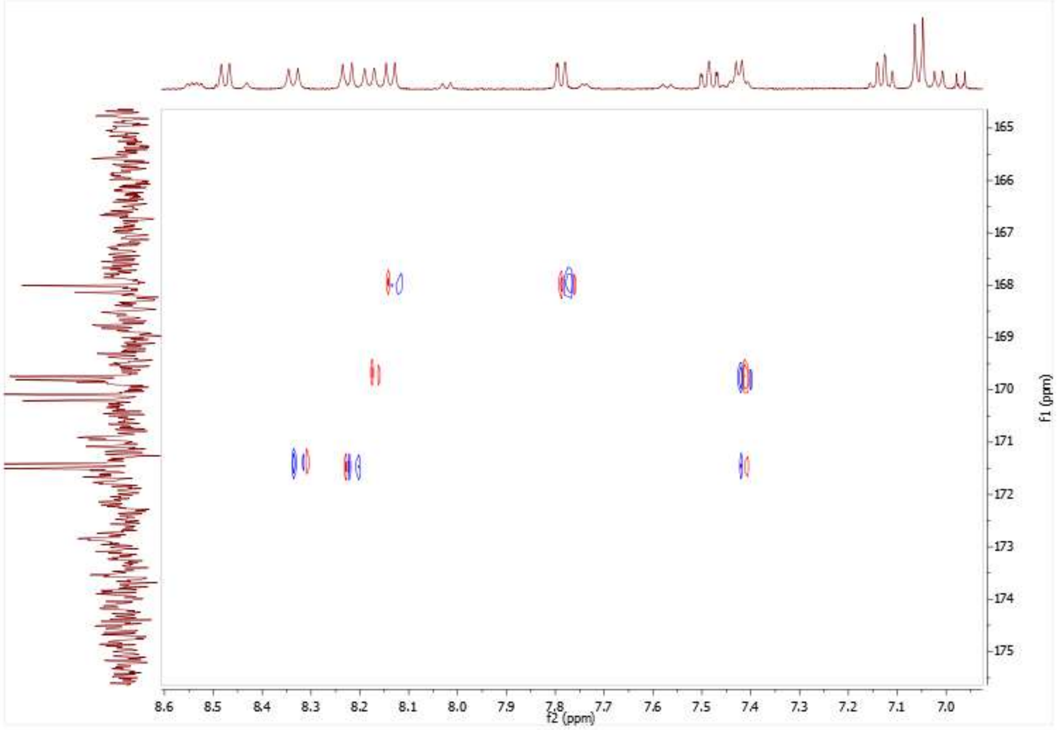

Figure S3.5. Expansion of a region of the HMBC spectrum

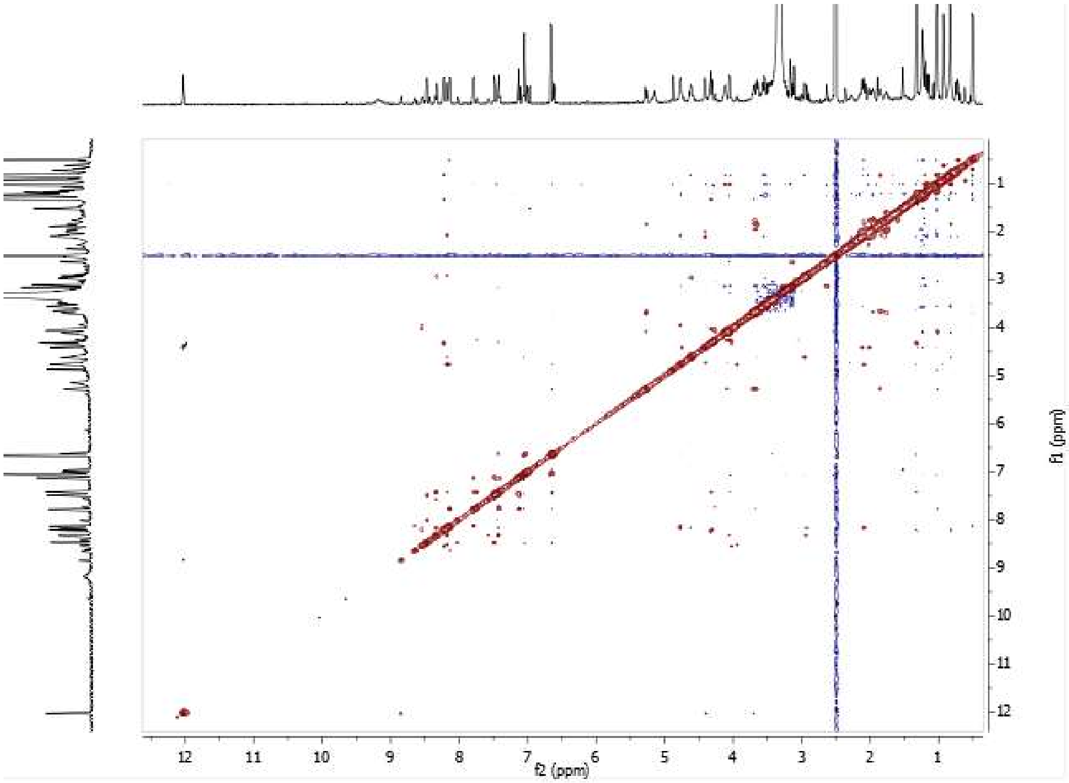

Figure S3.6. NOESY spectrum of cadophorin A

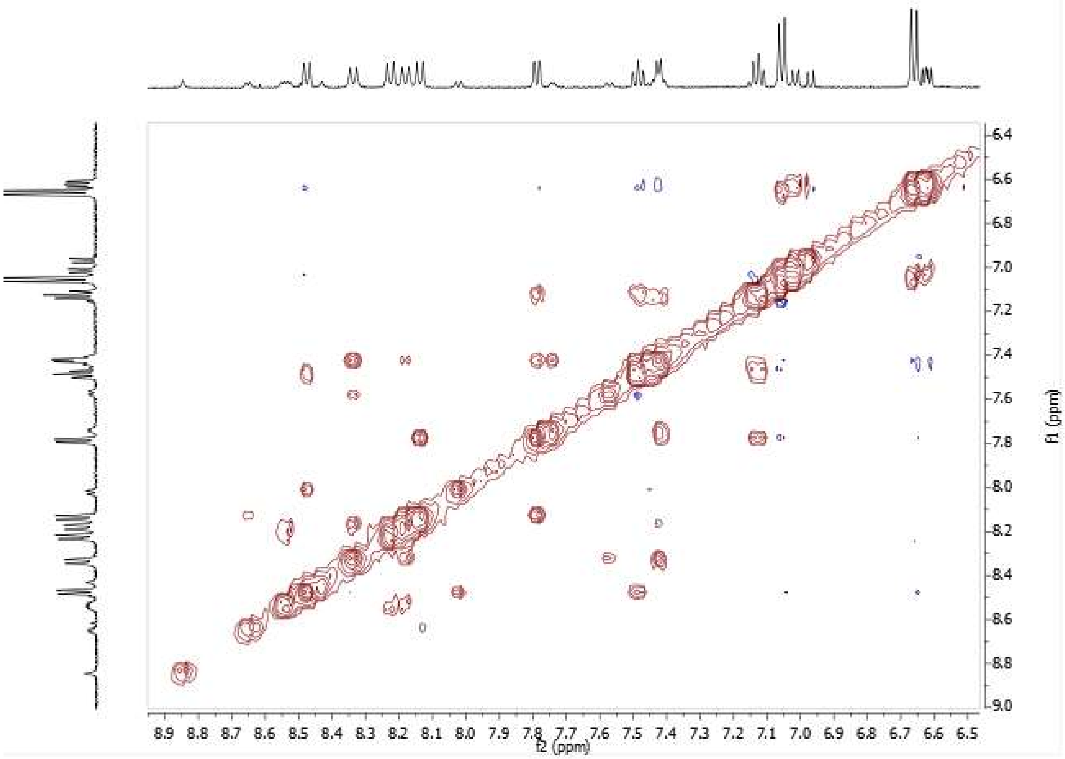

Figure S3.7. Expansion of the NOESY spectrum

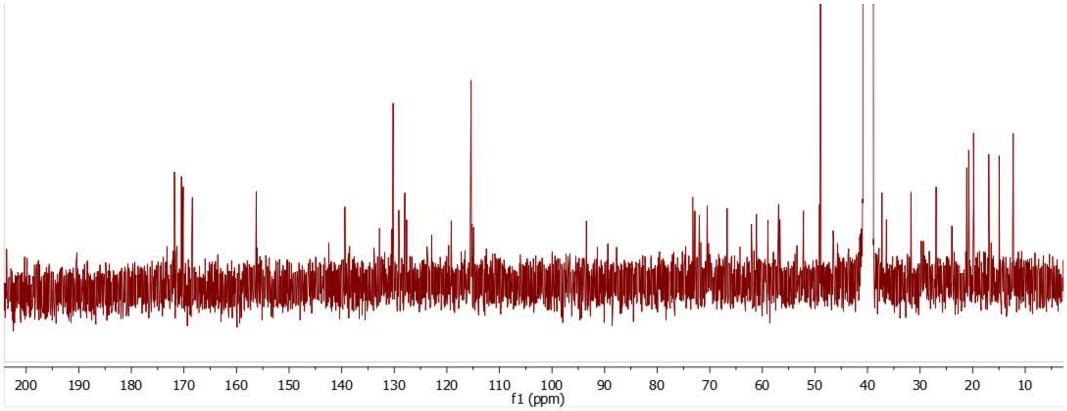

Figure S3.8. ^{13}C NMR spectrum of cadophorin A.

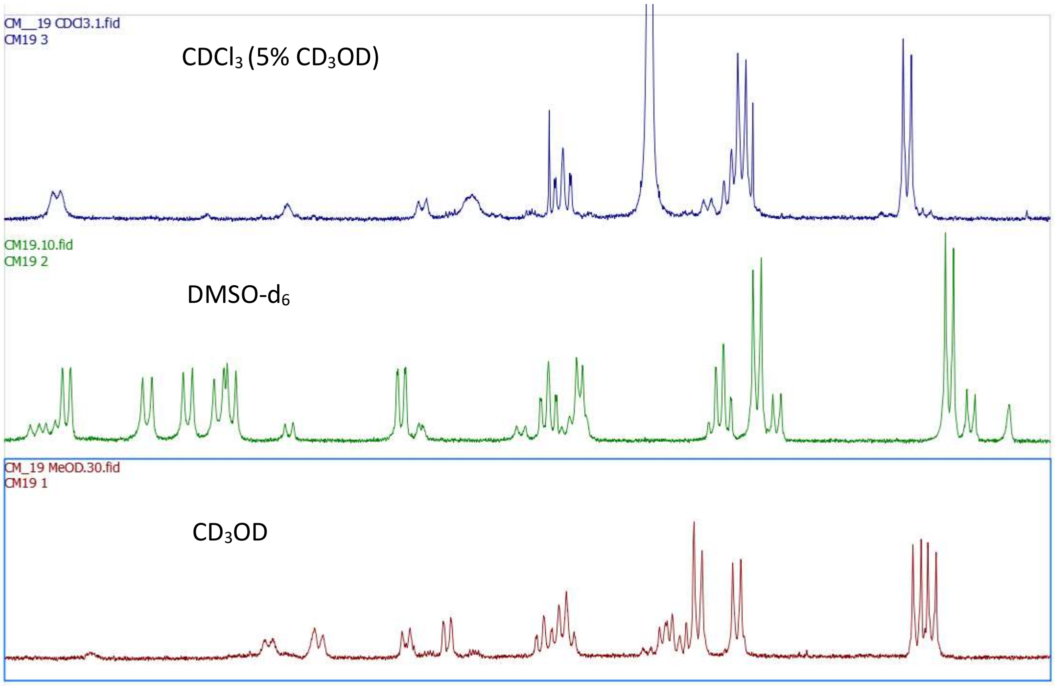

Figure S3.9. Comparison of the ^{1}H-NMR spectra of cadA in different NMR solvents

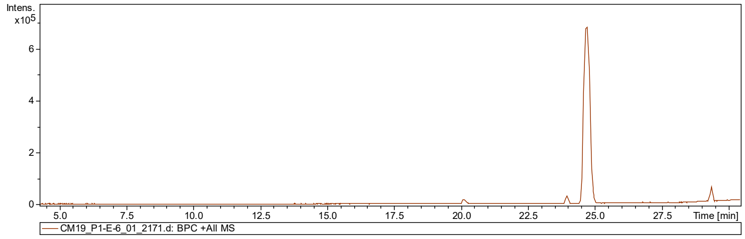

Figure S3.10. LCMS (BPC) chromatogram of cadophorin A

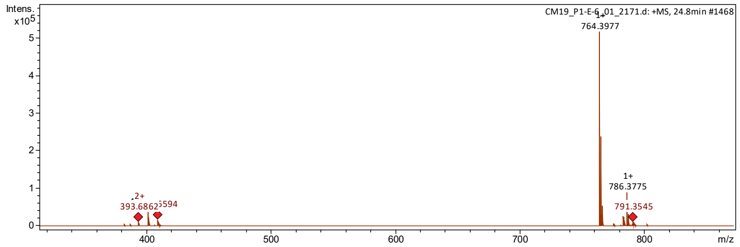

Figure S3.11. MS spectrum of cadophorin A

Meas. m/z	Ion Formula	m/z	err [ppm]	rdb	N-Rule	e⁻ Conf	mSigma
764.3977	C39H54N7O9	764.3977	0.0	17.0	ok	even	8.8

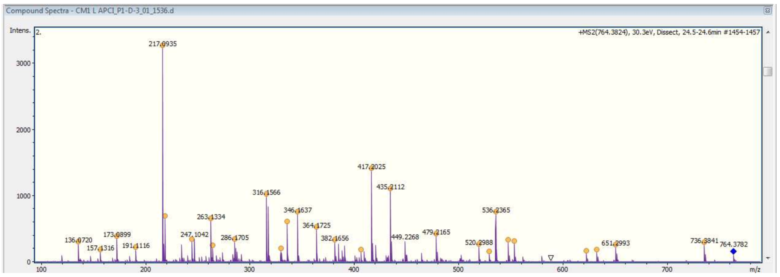

Figure S3.12. MS2 spectrum of m/z 764.38 (30 eV) (cadA). CE 30 eV.

Table S3.1. Fragments observed in the MS2 spectrum of cadophorin A

Fragment	m/z	Ion Formula	Error (ppm)	Assignment
	764.3983	$C_{39}H_{54}N_7O_9$	0.7	$[M+H]^+$
	736.4020	$C_{39}H_{54}N_7O_8$	1.1	$[M+H-CO]^+$
$Serie^{1,2}$	Ile-Pro-ATA-Val-Tyr-Thr-Ala			
$b_3^{1,2}$	330.1806	$C_{18}H_{24}N_3O_3$	2.0	$[Ile-Pro-ATA]^+$
$[b_2^{1,2}- CO]^+$	183.1479	$C_{10}H_{19}N_2O$	7.2	$[Ile-Pro-CO]^+$
$Serie^{2,3}$	Pro-ATA-Val-Tyr-Thr-Ala-Ile			
$b_6^{2,3}$	651.3179	$C_{33}H_{43}N_6O_8$	1.9	$[Pro-ATA-Val-Tyr-Thr-Ala]^+$
$[b_6^{2,3}-H_2O]^+$	633.3023	$C_{33}H_{41}N_6O_7$	1.2	$[Pro-ATA-Val-Tyr-Thr-Ala- H_2O]^+$
$[b_6^{2,3}-CO]^+$	623.3189	$C_{32}H_{43}N_6O_7$	1	$[Pro-ATA-Val-Tyr-Thr-Ala- CO]^+$
$b_5^{2,3}$	580.2768	$C_{30}H_{38}N_5O_7$	4.7	$[Pro-ATA-Val-Tyr-Thr]^+$
$b_4^{2,3}$	479.2294	$C_{26}H_{31}N_4O_5$	1.0	$[Pro-ATA-Val-Tyr]^+$
$b_3^{2,3}$	316.1653	$C_{17}H_{22}N_3O_3$	0.8	$[Pro-ATA-Val]^+$
$b_2^{2,3}$	217.0977	$C_{12}H_{13}N_2O_2$	2.5	$[Pro-ATA]^+$
$Serie^{3,4}$	ATA-Val-Tyr-Thr-Ala-Ile-Pro			
$b_6^{3,4}$	667.3453	$C_{34}H_{47}N_6O_8$	0.5	$[ATA-Val-Tyr-Thr-Ala-Ile]^+$
$b_5^{3,4}$	554.2620	$C_{28}H_{36}N_5O_7$	1.9	$[ATA-Val-Tyr-Thr-Ala]^+$
$b_4^{3,4}$	483.2224	$C_{25}H_{31}N_4O_6$	3	$[ATA-Val-Tyr-Thr]^+$
$b_3^{3,4}$	382.1745	$C_{21}H_{24}N_3O_4$	4.3	$[ATA-Val-Tyr]^+$
$b_2^{3,4}$	219.1127	$C_{12}H_{15}N_2O_2$	0.3	$[ATA-Val]^+$
$[b_2^{3,4} - CO]^+$	191.1163	$C_{11}H_{15}N_2O$	8.5	$[ATA-Val- CO]^+$
$Serie^{4,5}$	Val-Tyr-Thr-Ala-Ile-Pro-ATA			
$b_5^{4,5}$	548.3066	$C_{27}H_{42}N_5O_7$	2.2	$[Val-Tyr-Thr-Ala-Ile]^+$
$[b_5^{4,5}-H_2O]^+$	530.2978	$C_{27}H_{40}N_5O_6$	1.0	$[Val-Tyr-Thr-Ala-Ile -H_2O]^+$
$[b_5^{4,5}-CO]^+$	520.3098	$C_{26}H_{42}N_5O_6$	6.1	$[Val-Tyr-Thr-Ala-Ile -CO]^+$
$b_4^{4,5}$	435.2222	$C_{21}H_{31}N_4O_6$	3.6	$[Val-Tyr-Thr-Ala]^+$
$[b_4^{4,5}-H_2O]^+$	417.2112	$C_{21}H_{29}N_4O_5$	5.0	$[Val-Tyr-Thr-Ala -H_2O]^+$
$[b_4^{4,5}-CO]^+$	407.2334	$C_{20}H_{31}N_4O_5$	0.7	$[Val-Tyr-Thr-Ala -CO]^+$
$b_3^{4,5}$	364.1840	$C_{18}H_{26}N_3O_5$	5.5	$[Val-Tyr-Thr]^+$
$b_2^{4,5}$	263.1390	$C_{14}H_{19}N_2O_3$	0.1	$[Val-Tyr]^+$
$[b_3^{4,5}-H_2O]^+$	245.1277	$C_{14}H_{17}N_2O_2$	4.7	$[Val-Tyr -H_2O]^+$
$Serie^{5,6}$	Tyr-Thr-Ala-Ile-Pro-ATA-Val			
$b_4^{5,6}$	449.2310	$C_{22}H_{33}N_4O_6$	1.3	$[Tyr-Thr-Ala-Ile]^+$
$b_3^{5,6}$	336.1486	$C_{16}H_{22}N_3O_5$	0.2	$[Tyr-Thr-Ala]^+$
$[b_1^{5,6}-CO]^+$	136.0725	$C_8H_{10}NO$	5.3	$[Tyr -CO]^+$

Serie[6,7]	Thr-Ala-Ile-Pro-ATA-Val-Tyr			
$b_3^{6,7}$	286.1763	$C_{25}H_{36}N_3O_4$	0.7	$[Thr\text{-}Ala\text{-}Ile]^+$
$b_2^{6,7}$	173.0924	$C_7H_{13}N_2O_3$	2.1	$[Thr\text{-}Ala]^+$
Serie[7,1]	Ala-Ile-Pro-ATA-Val-Tyr-Thr			
$b_4^{7,1}$	401.1279	$C_{21}H_{29}N_4O_4$	1.1	$[Ala\text{-}Ile\text{-}Pro\text{-}ATA]^+$
$b_2^{7,1}$	185.1283	$C_9H_{17}N_2O_2$	1.1	$[Ala\text{-}Ile]^+$
$[b_2^{7,1} - CO]^+$	157.1333	$C_2H_{17}N_2O$	1.8	$[Ala\text{-}Ile\text{- }CO]^+$

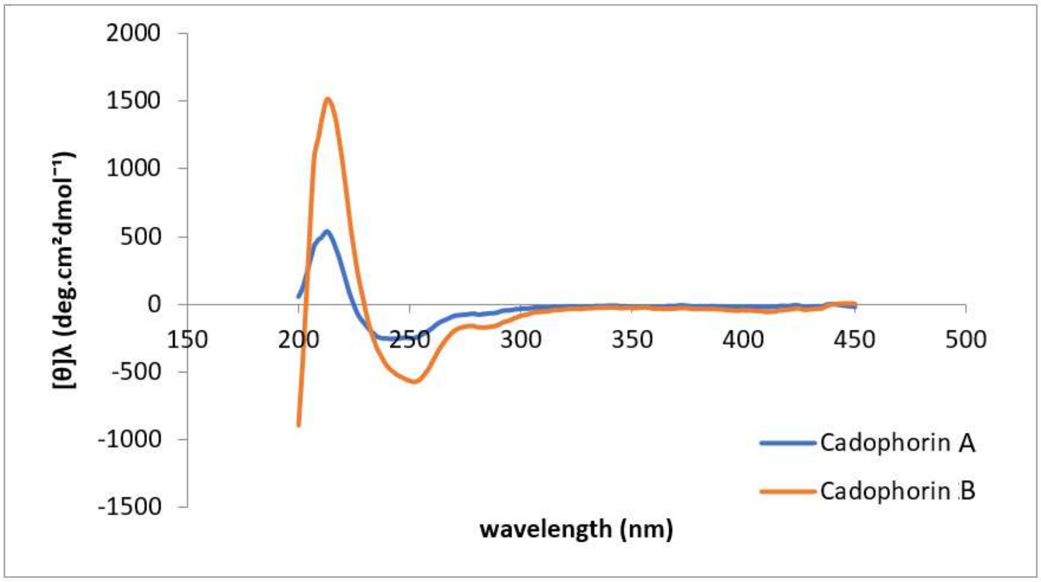

Figure S3.13. ECD spectra for cadophorin A and B.

Part 4. Spectroscopic data of cadophorin **B**

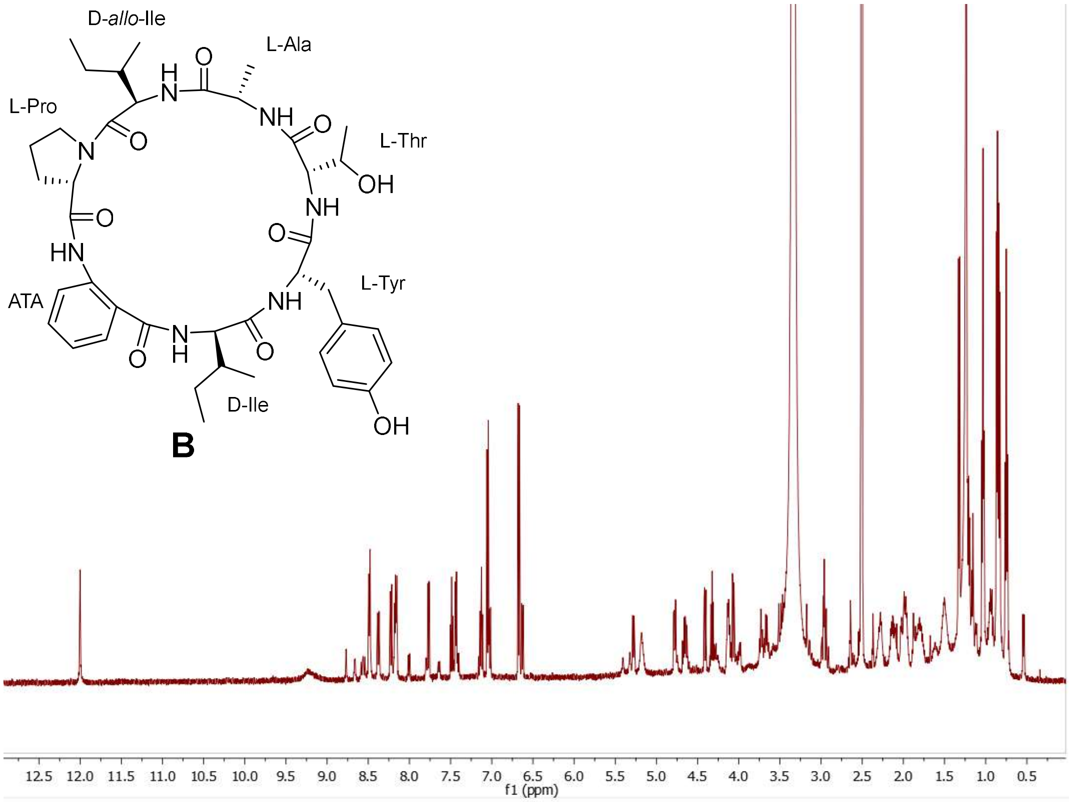

Figure S4.1. ^{1}H NMR spectrum of cadophorin B (DMSO-d_6)

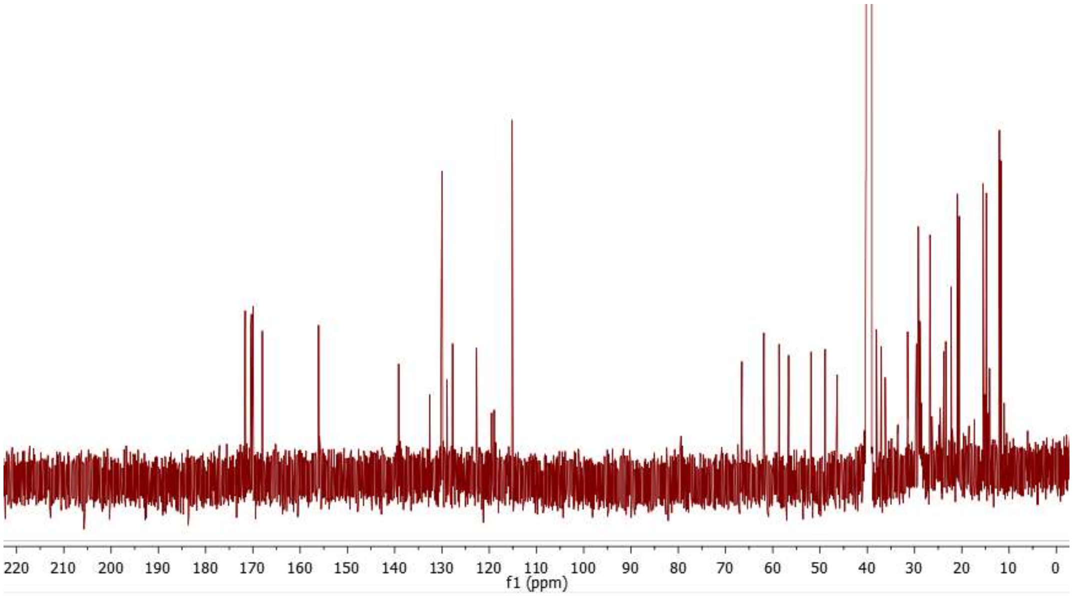

Figure S4.2. ^{13}C NMR of cadophorin B.

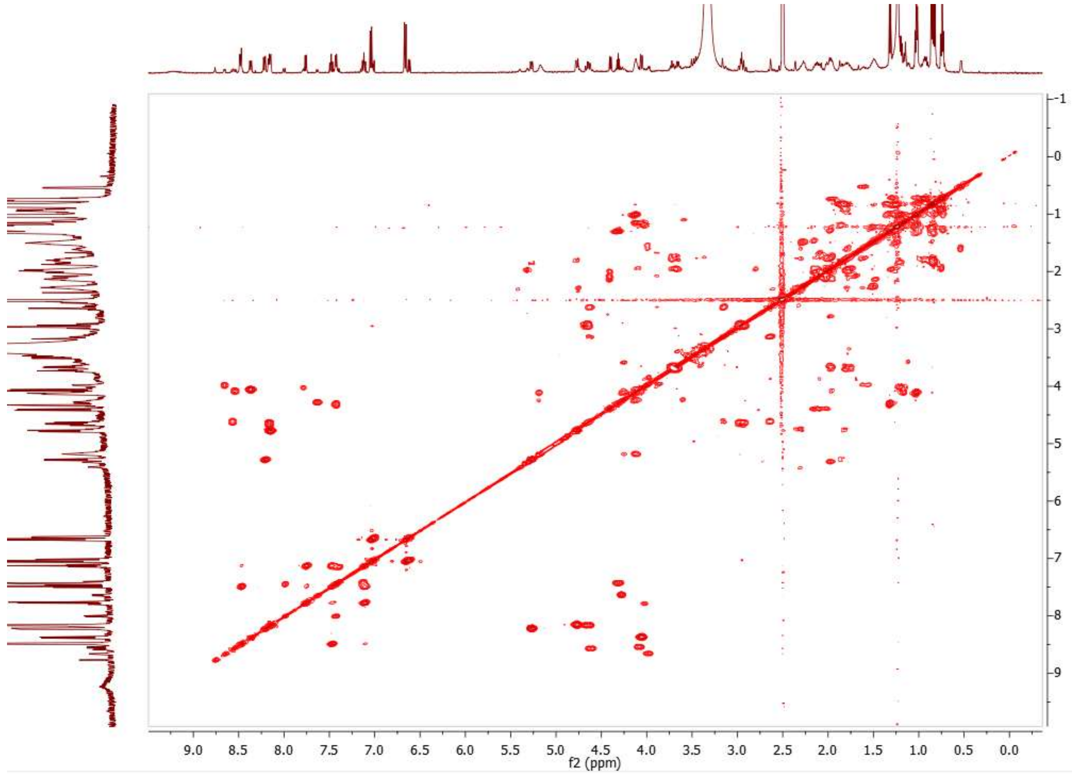

Figure S4.3. ^{1}H- ^{1}H COSY spectrum of cadophorin B

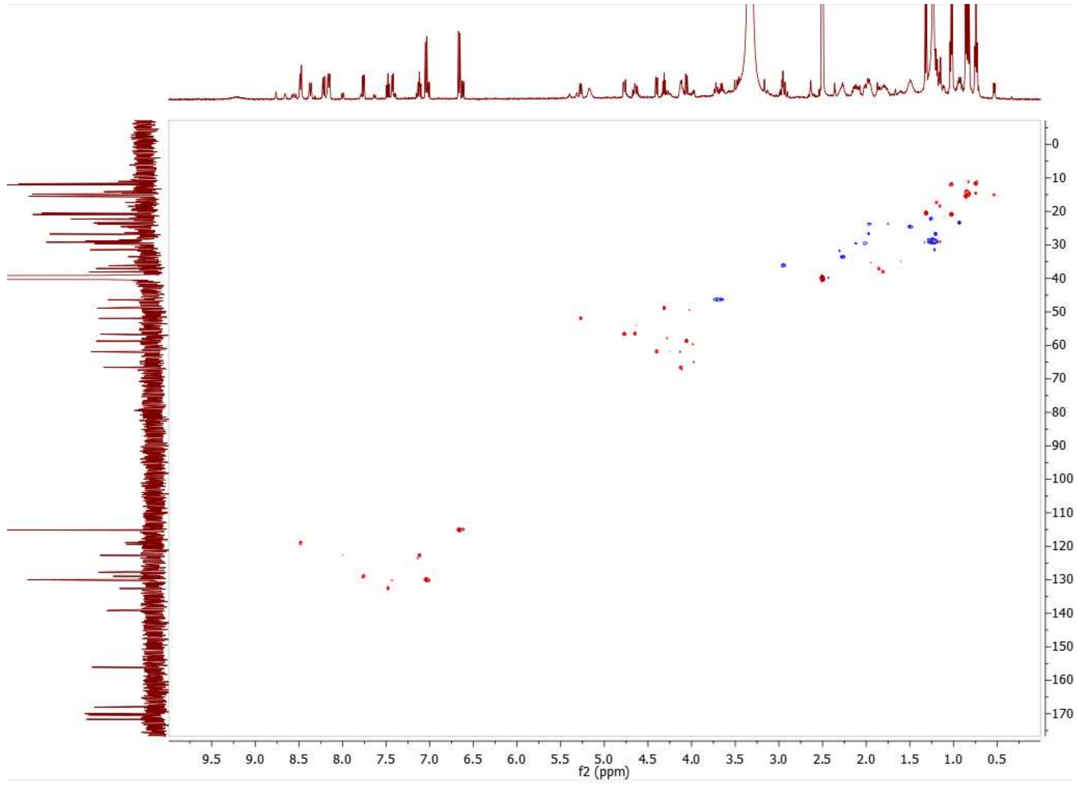

Figure S4.4. HSQC-DEPT spectrum of cadophorin B

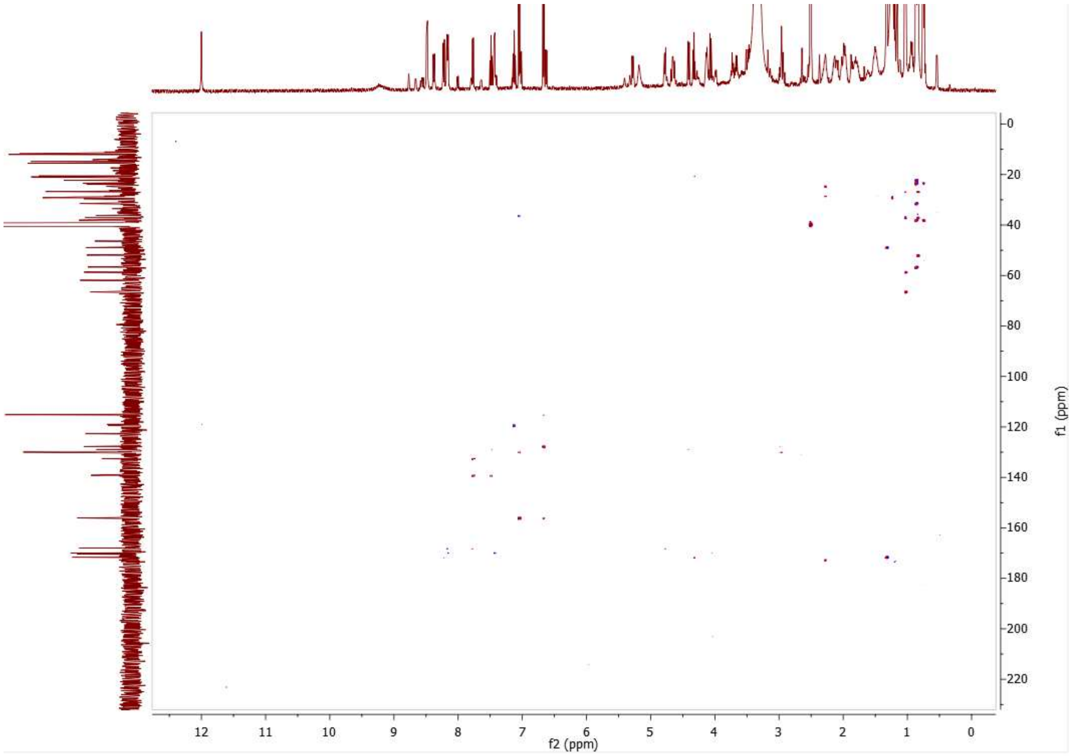

Figure S4.5. HMBC spectrum of cadophorin B

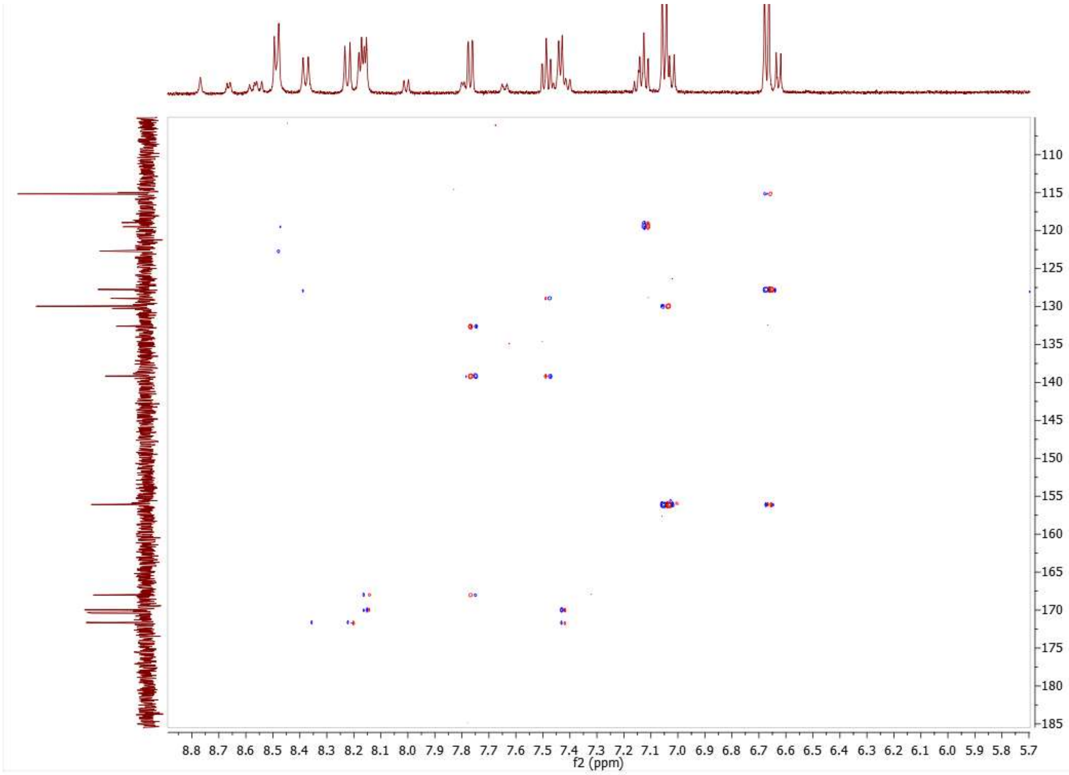

Figure S4.6. Expansion of the HMBC spectrum of cadophorin B

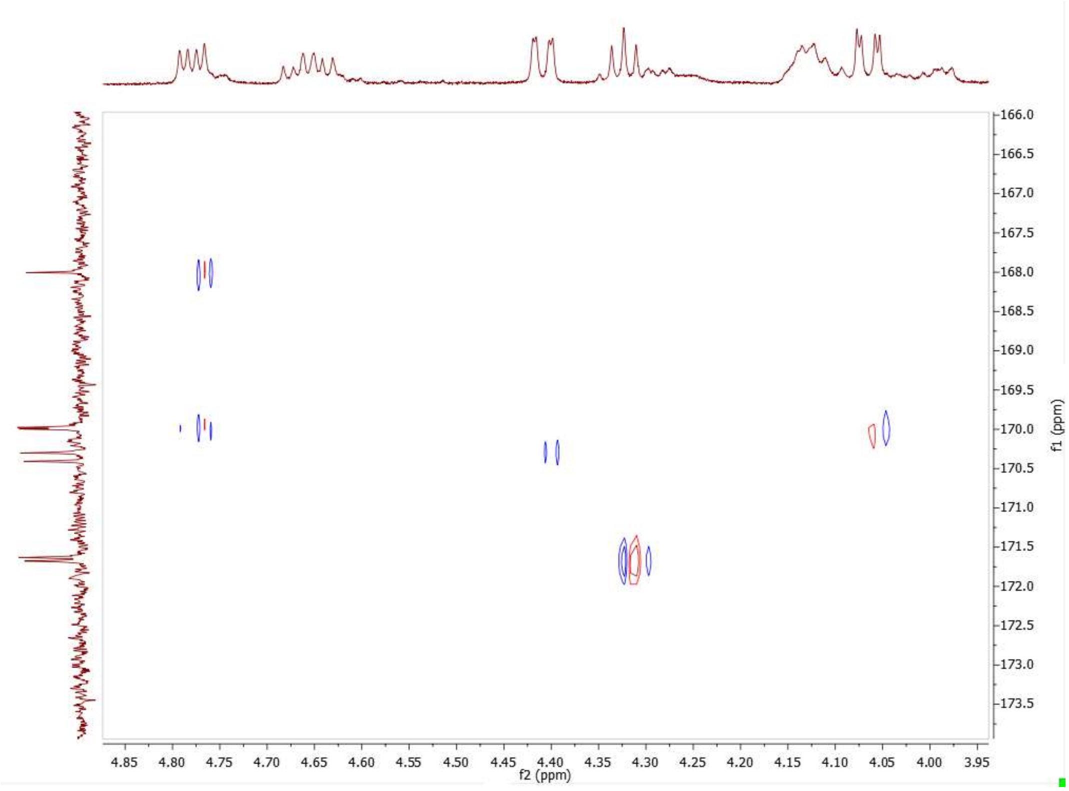

Figure S4.7. Expansion of the HMBC spectrum of cadophorin B

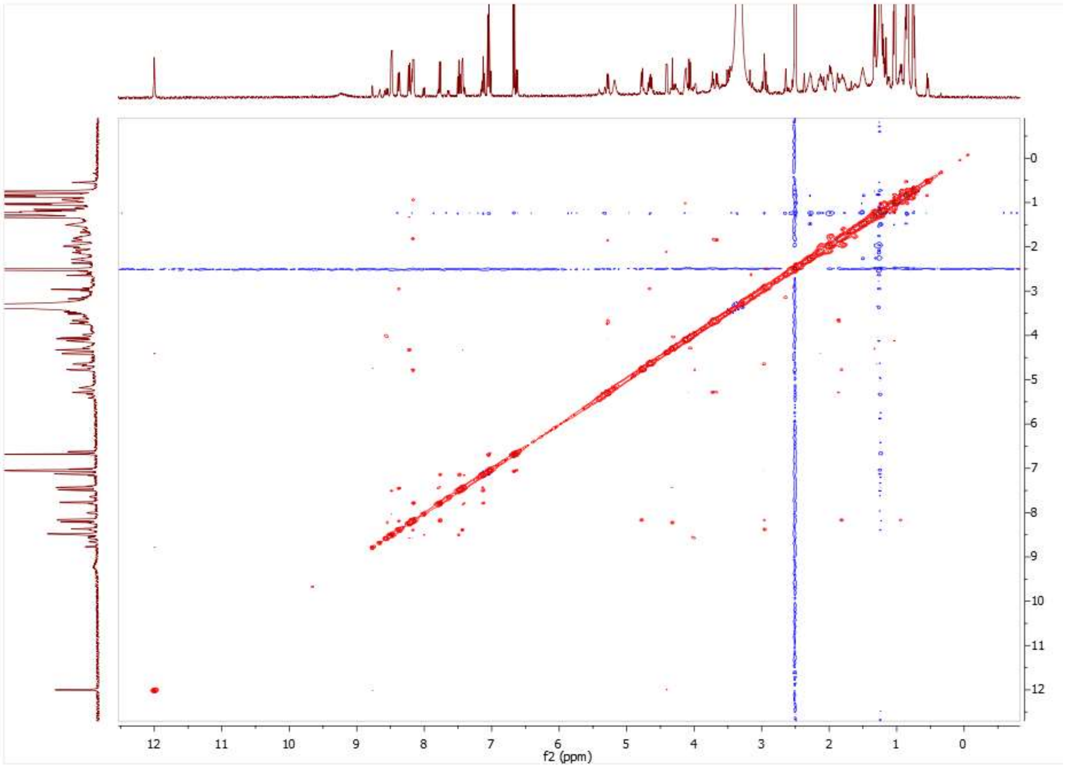

Figure S4.8. NOESY spectrum of cadophorin B.

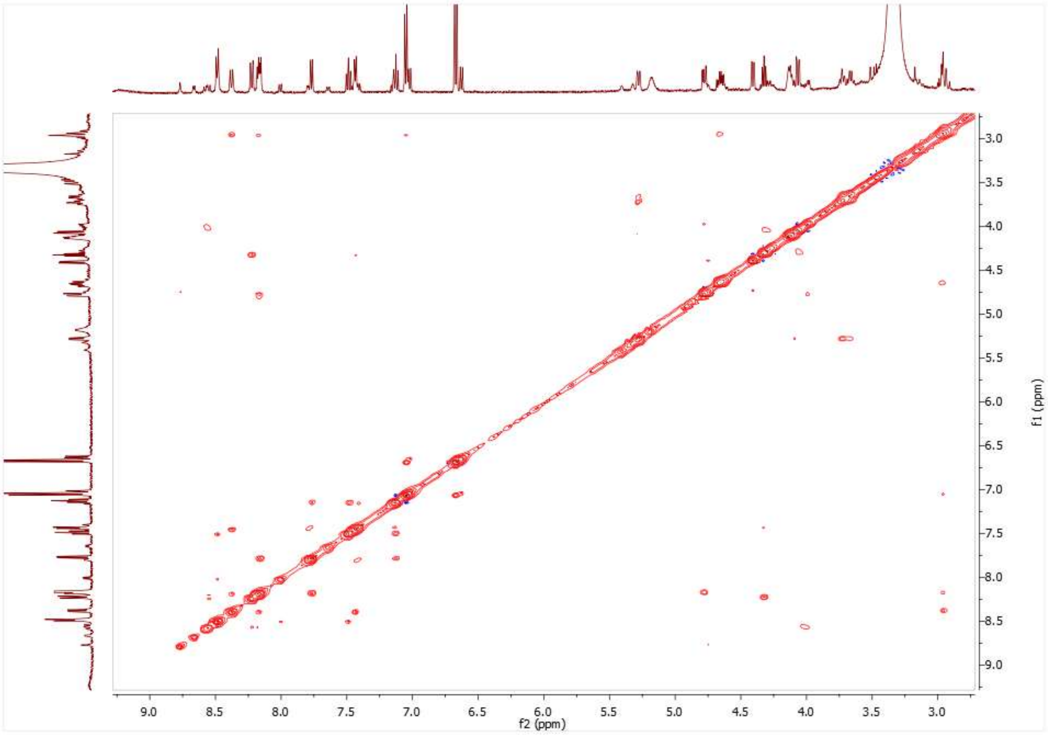

Figure S4.9. Expansion of the NOESY spectrum of cadophorin B.

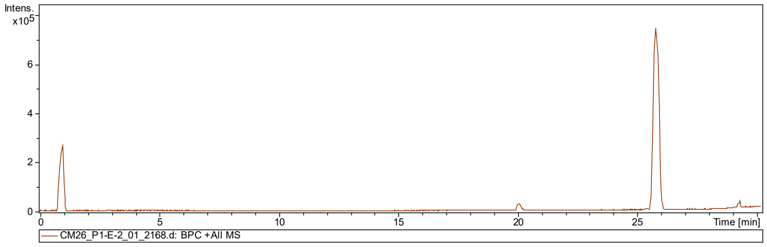

Figure S4.10. LCMS (BPC) chromatogram of cadophorin B

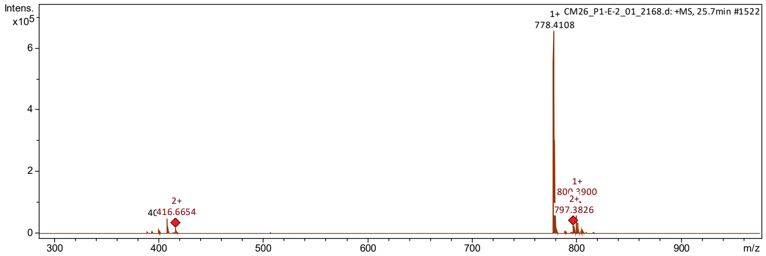

Figure S4.11. MS spectrum of cadophorin B

Meas. m/z	Ion Formula	m/z	err [ppm]	rdb	N-Rule	e^- Conf	mSigma
778.4108	C40H56N7O9	778.4134	3.3	17.0	ok	even	10.0

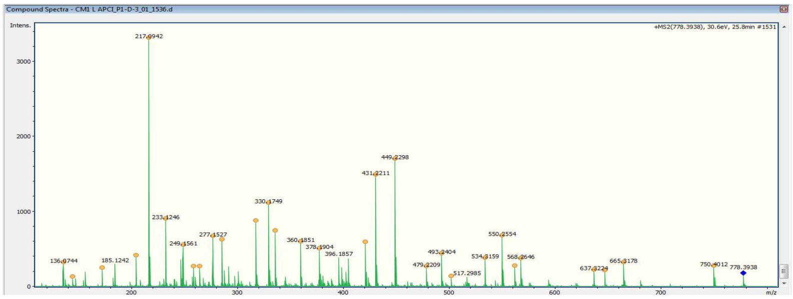

Figure S4.12. MS2 spectrum of m/z 778.4 (cadophorin B). CE 30 eV.

Table S4.1. Fragments observed in the MS2 spectrum of cadophorin B

Fragment	m/z	Ion Formula	Error (ppm)	Assignment
	778.4135	$C_{40}H_{56}N_7O_9$	0.1	$[M+H]^+$
	750.4163	$C_{39}H_{56}N_7O_8$	3.0	$[M+H-CO]^+$
$Serie^{1,2}$	Ile_1-Pro-Ata-Ile_2-Tyr-Thr-Ala			
$b_5^{1,2}$	606.3234	$C_{33}H_{44}N_5O_6$	8.6	$[Ile_1\text{-Pro-Ata-}Ile_2\text{-Tyr}]^+$
$b_3^{1,2}$	330.1798	$C_{18}H_{24}N_3O_3$	4.3	$[Ile_1\text{-Pro-ATA}]^+$
$b_2^{1,2}$	211.1429	$C_{11}H_{19}N_2O_2$	5.9	$[Ile_1\text{-Pro}]^+$
$Serie^{2,3}$	Pro-ATA-Ile_2-Tyr-Thr-Ala-Ile_1			
$b_6^{2,3}$	665.3274	$C_{34}H_{45}N_6O_8$	2.9	$[\text{Pro-ATA-}Ile_2\text{-Tyr-Thr-Ala}]^+$
$[b_6^{2,3}-H_2O]^+$	647.3147	$C_{34}H_{43}N_6O_7$	6.2	$[\text{Pro-ATA-}Ile_2\text{-Tyr-Thr-Ala}– H_2O]^+$
$[b_6^{2,3}-CO]^+$	637.3337	$C_{33}H_{45}N_6O_7$	1.1	$[\text{Pro-ATA-}Ile_2\text{-Tyr-Thr-Ala}– CO]^+$
$b_5^{2,3}$	594.2915	$C_{31}H_{40}N_5O_7$	1.2	$[\text{Pro-ATA-}Ile_2\text{-Tyr-Thr}]^+$
$b_4^{2,3}$	493.2416	$C_{27}H_{33}N_4O_5$	6.0	$[\text{Pro-ATA-}Ile_2\text{-Tyr}]^+$
$[b_4^{2,3}-CO]^+$	465.2465	$C_{26}H_{33}N_4O_4$	6.8	$[\text{Pro-ATA-}Ile_2\text{-Tyr} – CO]^+$
$b_3^{2,3}$	330.1798	$C_{18}H_{24}N_3O_3$	4.3	$[\text{Pro-ATA-}Ile_2]^+$
$b_2^{2,3}$	217.0961	$C_{12}H_{13}N_2O_2$	4.8	$[\text{Pro-ATA}]^+$
$Serie^{3,4}$	ATA-Ile_2-Tyr-Thr-Ala-Ile_1-Pro			
$b_6^{3,4}$	681.3584	$C_{35}H_{49}N_6O_8$	3.3	$[\text{ATA-}Ile_2\text{-Tyr-Thr-Ala-}Ile_1]^+$
$b_5^{3,4}$	568.2741	$C_{29}H_{38}N_5O_7$	4.4	$[\text{ATA-}Ile_2\text{-Tyr-Thr-Ala}]^+$
$[b_5^{3,4}-H_2O]^+$	550.2638	$C_{29}H_{36}N_5O_6$	3.9	$[\text{ATA-}Ile_2\text{-Tyr-Thr-Ala} – H_2O]^+$
$b_4^{3,4}$	497.2385	$C_{26}H_{33}N_4O_6$	1.9	$[\text{ATA-}Ile_2\text{-Tyr-Thr}]^+$
$[b_4^{3,4}-H_2O]^+$	479.2267	$C_{26}H_{31}N_4O_5$	4.9	$[\text{ATA-}Ile_2\text{-Tyr-Thr} – H_2O]^+$
$b_2^{3,4}$	233.1274	$C_{13}H_{17}N_2O_2$	4.7	$[\text{ATA-}Ile_2]^+$
$[b_2^{3,4}-CO]^+$	205.1325	$C_{12}H_{17}N_2O$	5.3	$[\text{ATA-}Ile_2 – CO]^+$
$b_6^{3,4}$	681.3584	$C_{35}H_{49}N_6O_8$	3.3	$[\text{ATA-}Ile_2\text{-Tyr-Thr-Ala-}Ile_1]^+$
$Serie^{4,5}$	Ile_2-Tyr-Thr-Ala-Ile_1-Pro-ATA			
$b_5^{4,5}$	562.3224	$C_{28}H_{44}N_5O_7$	2	$[Ile_2\text{-Tyr-Thr-Ala-}Ile_1]^+$
$[b_5^{4,5}-H_2O]^+$	544.3122	$C_{28}H_{42}N_5O_6$	1.4	$[Ile_2\text{-Tyr-Thr-Ala-}Ile_1– H_2O]^+$
$[b_5^{4,5}-CO]^+$	532.3247	$C_{27}H_{44}N_5O_6$	7.3	$[Ile_2\text{-Tyr-Thr-Ala-}Ile_1– CO]^+$
$b_4^{4,5}$	449.2376	$C_{22}H_{33}N_4O_6$	4.1	$[Ile_2\text{-Tyr-Thr-Ala}]^+$
$[b_4^{4,5}-H_2O]^+$	431.2284	$C_{22}H_{31}N_4O_5$	1.2	$[Ile_2\text{-Tyr-Thr-Ala} – H_2O]^+$
$[b_4^{4,5}-CO]^+$	421.2414	$C_{21}H_{33}N_4O_5$	7	$[Ile_2\text{-Tyr-Thr-Ala} – CO]^+$
$b_3^{4,5}$	378.2006	$C_{19}H_{28}N_3O_5$	4.5	$[Ile_2\text{-Tyr-Thr}]^+$
$[b_3^{4,5}-H_2O]^+$	360.1889	$C_{19}H_{26}N_3O_4$	8	$[Ile_2\text{-Tyr-Thr} – H_2O]^+$
$[b_3^{4,5}-CO]^+$	350.2041	$C_{18}H_{28}N_3O_4$	9.5	$[Ile_2\text{-Tyr-Thr} – CO]^+$

$b_2^{4,5}$	277.1553	$C_{15}H_{21}N_2O_3$	5.1	$[Ile_2\text{-}Tyr]^+$
$[b_3^{4,5}\text{-}H_2O]^+$	259.1446	$C_{15}H_{19}N_2O_2$	2	$[Ile_2\text{-}Tyr - H_2O]^+$
$[b_3^{4,5}\text{-}CO]^+$	249.1584	$C_{14}H_{21}N_2O_2$	5.5	$[Ile_2\text{-}Tyr - CO]^+$
Serie^{5,6}	Tyr-Thr-Ala-Ile$_1$-Pro-ATA-Ile$_2$			
$b_3^{5,6}$	336.1514	$C_{16}H_{22}N_3O_5$	4.3	[Tyr-Thr-Ala]$^+$
$[b_3^{5,6} - H_2O]^+$	318.1431	$C_{16}H_{20}N_3O_4$	5.4	[Tyr-Thr-Ala – H_2O]$^+$
$b_2^{5,6}$	265.1190	$C_{13}H_{17}N_2O_4$	2.8	[Tyr-Thr]$^+$
$[b_1^{5,6}\text{-}CO]^+$	136.0744	$C_8H_{10}NO$	9.2	[Tyr – CO]$^+$
Serie ^{6,7}	Thr-Ala-Ile$_1$-Pro-ATA-Ile$_2$-Tyr			
$b_5^{6,7}$	502.2621	$C_{25}H_{36}N_5O_6$	7.8	[Thr-Ala-Ile$_1$-Pro-ATA]$^+$
$b_3^{6,7}$	286.1758	$C_{13}H_{24}N_3O_4$	1.3	[Thr-Ala-Ile$_1$]$^+$
$b_2^{6,7}$	173.0910	$C_7H_{13}N_2O_3$	6	[Thr-Ala]$^+$
$[b_2^{6,7}\text{-}CO]^+$	145.0958	$C_6H_{13}N_2O_2$	9.5	[Thr-Ala – CO]$^+$
Serie^{7,1}	Ala-Ile$_1$-Pro-Ata-Ile$_2$-Tyr-Thr			
$b_4^{7,1}$	401.2079	$C_{21}H_{29}N_4O_4$		[Ala-Ile$_1$-Pro-Ata]$^+$
$b_2^{7,1}$	185.1278	$C_9H_{17}N_2O_2$	3.7	[Ala-Ile$_1$]$^+$
$[b_2^{7,1}\text{-}CO]^+$	157.1336	$C_8H_{17}N_2O$	0.4	[Ala-Ile$_1$ – CO]$^+$

Part 5

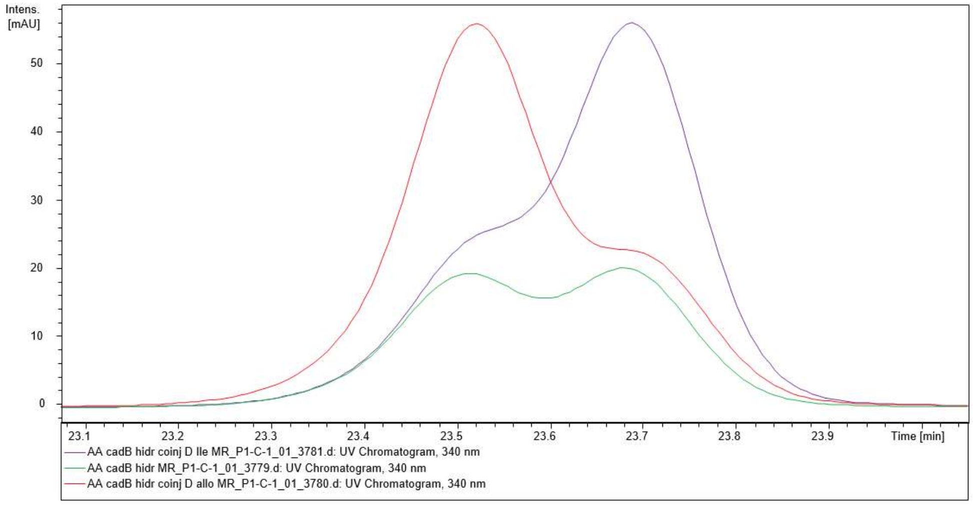

Figure 5.1. Comparison of the retention times of the L-FDVA derivatives of D-Ile, D-*allo*-Ile present in cadB to co-injected standards of L-FDVA derivatives of D-Ile and D-*allo*-Ile.

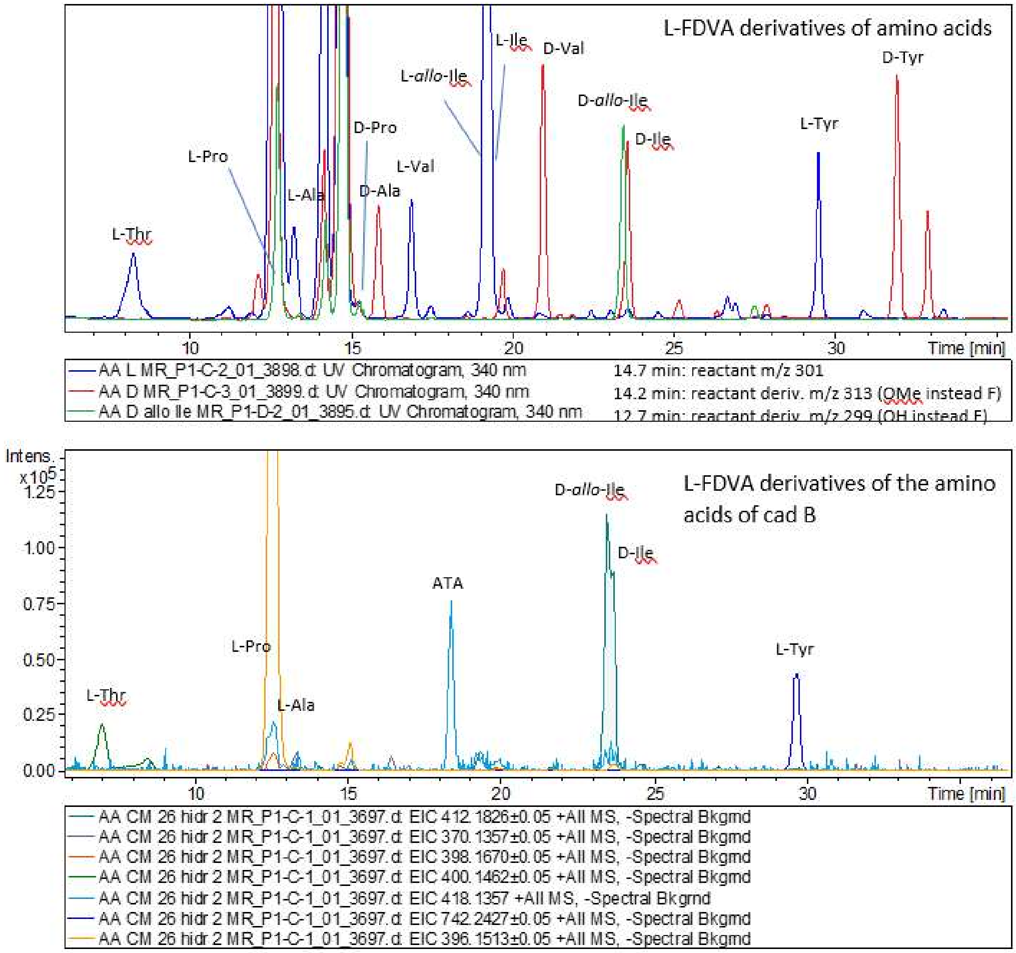

Identification was based on retention times, HR m/z, MS2 spectra and UV (340 nm)

Figure 5.2. Comparison of the retention times of the L-FDVA derivatives of L- and D- amino acids and the amino acids present in cadB.

Part 6.

Figure S6.1. MS spectra of cadB with post-column *in source* addition of metal salt solutions

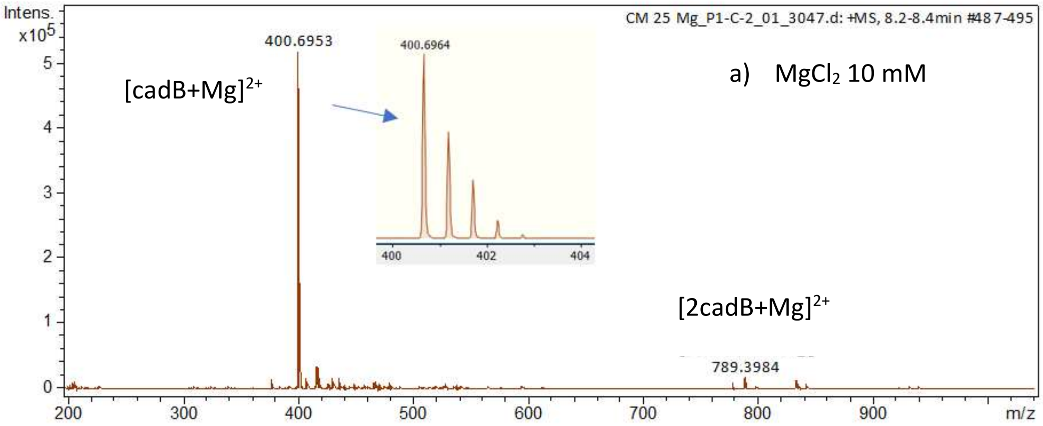

Meas. m/z	Ion Formula	m/z	err [ppm]
400.6964	C40H55MgN7O9	400.6950	-3.4

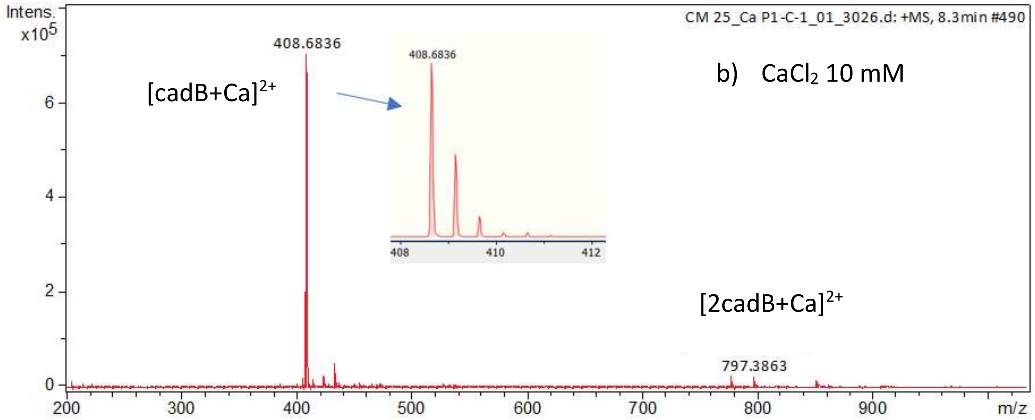

Meas. m/z	Ion Formula	m/z	err [ppm]
408.6836	C40H55CaN7O9	408.6838	0.6

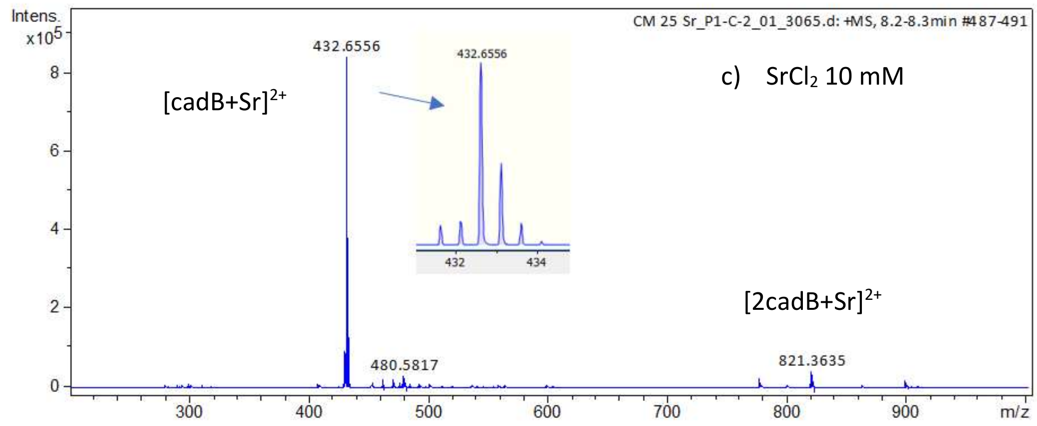

Meas. m/z	Ion Formula	m/z	err [ppm]
432.6556	C40H55N7O9Sr	432.6553	-0.2

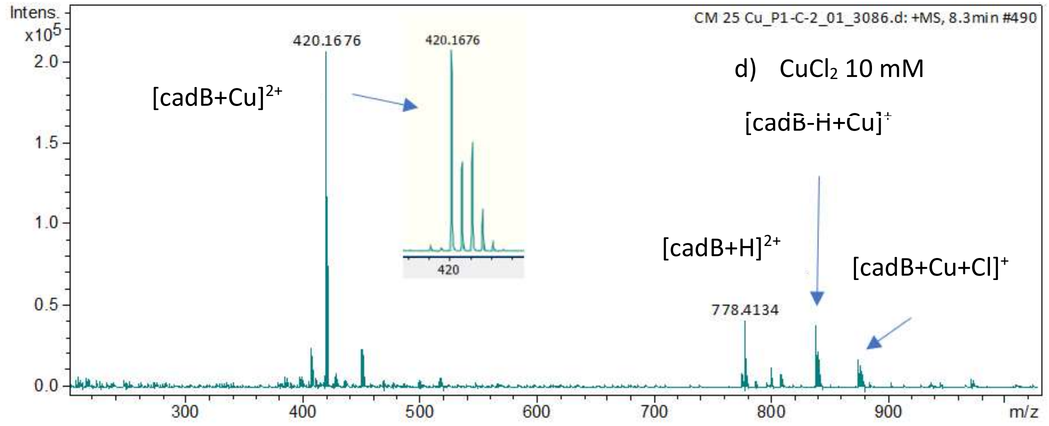

Meas. m/z	Ion Formula	m/z	err [ppm]
420.1676	C40H55CuN7O9	420.1673	0.7

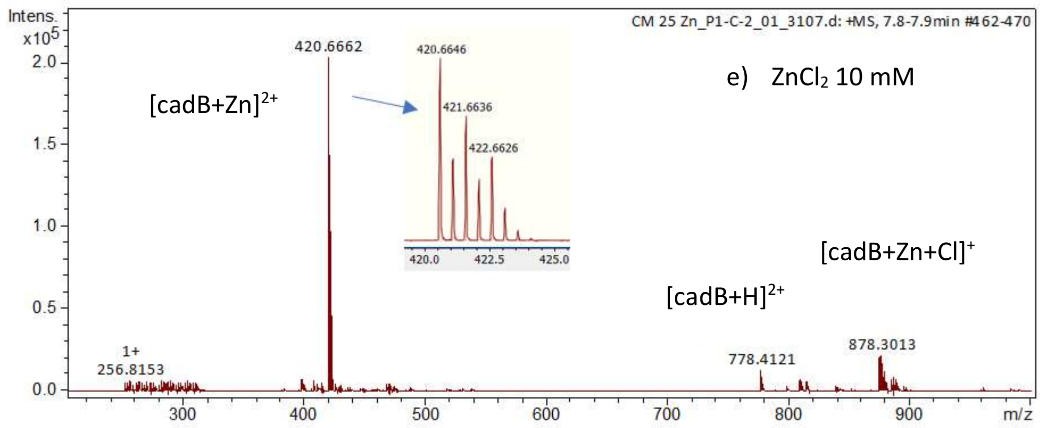

Meas. m/z	Ion Formula	m/z	err [ppm]
420.6662	C40H55N7O9Zn	420.6671	2.1

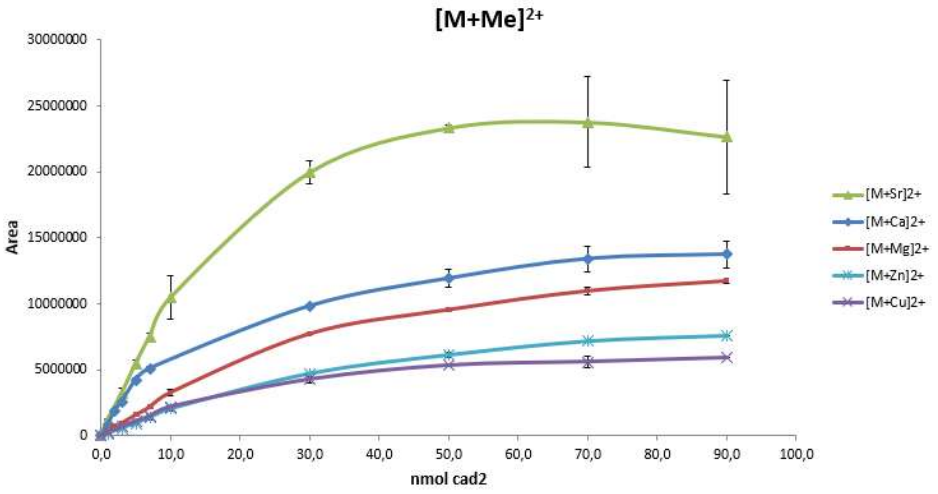

Figure S6.2. Relative abundances of the signals corresponding to $[M+Me]^{2+}$ vs amount of cadB.

Part 7. Computational methods

Complexes $[M+Me]^{2+}$ (Me= Mg, Ca, Sr, Cu, Zn) were optimized by DFT method using Gaussian 09 [1]. Frequency calculations were performed to characterize stationary points. Geometries of neutral and doubly charged molecules were optimized at the B3LYP hybrid density functional level of theory (DFT) [2,3] using the 6–31G(d) basis set for C, H, O, N, Mg, Ca, Cu, Zn, and LANL2DZ for Sr atoms. These bases set featuring polarized orbitals proved to be well suited for the investigation of similar systems in previous studies [4-6]. The optimized structures were characterized by harmonic frequency analysis as local minima (all frequencies real). Corrections for zero-point vibrational energy were included using the same level of theory.

All energies expressed as $\Delta H°$ are relative enthalpies at 0 K; those expressed as $\Delta G°$ are relative free energies at 298 K. Calculations were performed at the Centro de Cómputos de Alto Rendimiento (CeCAR, FCEyN, UBA).

[1] Gaussian 09, Revision A.02, Frisch M. J., Trucks G. W., Schlegel H. B., Scuseria G. E., Robb M. A., Cheeseman J. R., Scalmani G., Barone V., Petersson G. A., Nakatsuji H., X. Li, Caricato M., Marenich A., Bloino J., Janesko B. G., Gomperts R., Mennucci B., Hratchian H. P., Ortiz J. V., Izmaylov A. F., Sonnenberg J. L., Williams-Young D., Ding F., Lipparini F., Egidi F., Goings J., Peng B., Petrone A., Henderson T., Ranasinghe D., Zakrzewski V. G., Gao J., Rega N., Zheng G., Liang W., Hada M., Ehara M., Toyota K., Fukuda R., Hasegawa J., Ishida M., Nakajima T., Honda Y., Kitao O., Nakai H., Vreven T., Throssell K., Montgomery J. A., Jr., Peralta J. E., Ogliaro F., M., Heyd J. J., Brothers E., Kudin K. N., Staroverov V. N., Keith T., Kobayashi B. R., Normand J., Raghavachari K., Rendell A., Burant J. C., Iyengar S. S., Tomasi J., Cossi M., Millam J. M., Klene M., Adamo C., Cammi R., Ochterski J. W., Martin R. L., Morokuma K., Farkas O., Foresman J. B., Fox D. J., Gaussian, Inc., Wallingford CT (2016).
[2] C. Lee, W. Yang, R.G. Parr, Phys. Rev. B. 37, 785-789 (1988).
[3] A.D. Becke, J. Chem. Phys. 98, 5648-5652 (1993).
[4] I. Nicolás, M. Castro, J. Phys. Chem. A. 110, 4564-4573 (2006).
[5] A.E.M. Crotti, E.S. Bronze-Uhle, P.G.B.D. Nascimento, P.M. Donate, S.E. Galembeck, R. Vessecchia, N.P. Lopes, J. Mass. Spectrom. 44, 1733-1741 (2009).
[6] F. Shahangia, A.N. Chermahini, H. Farrokhpoura, A.Teimouri, RSC Adv. 5, 2305-2317 (2015).

a

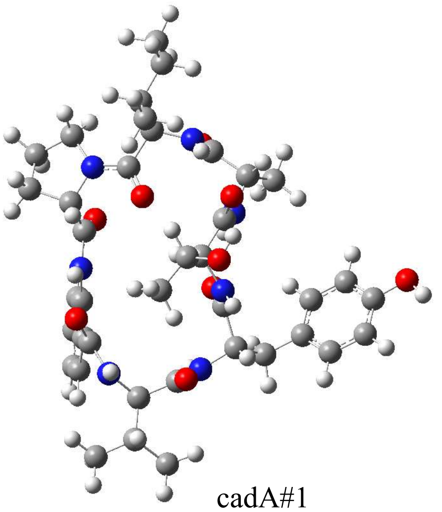

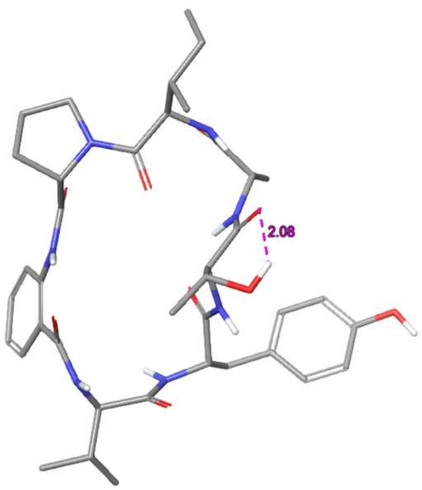

cadA#1

(H bond between HO of Thr and CO of Thr)

b

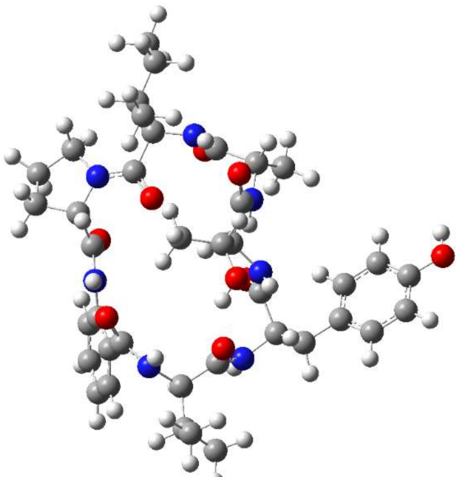

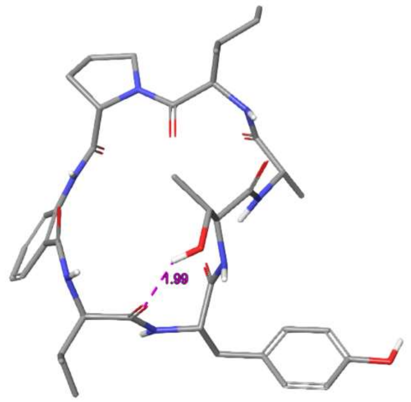

cadA#2

(H bond between HO of Thr and CO of Val)

c

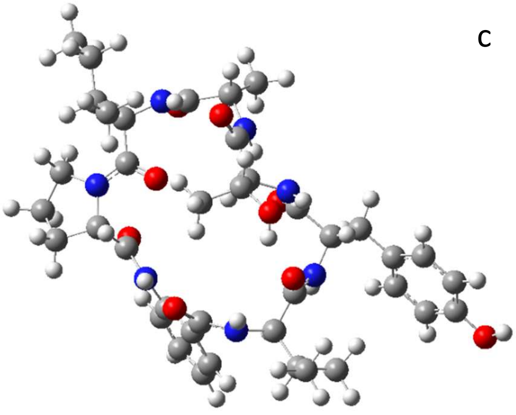

cadA#3

(different orientation of Tyr)

conformer	B3LYP 6-31G*/PCM DMSO (kcal/mol)
cadA#1 (a)	0.000
cadA#2 (b)	1.468
cadA#3 (c)	1.970

Figure S7.1. Lower energy conformers of cadophorin A.

Table S7.1. Geometrical parameters and charge distribution for cadA-Me complexes calculated at the B3LYP level of theory, Me= Mg^{2+}, Ca^{2+}, Sr^{2+}, Zn^{2+} and Cu^{2+}

complex	Metal centered geometry
$[cadA+Mg]^{2+}$	trigonal bypiramidal
$[cadA+Ca]^{2+}$	distorted octahedral
$[cadA+Sr]^{2+}$	distorted octahedral
$[cadA+Zn]^{2+}$	trigonal bypiramidal
$[cadA+Cu]^{2+}$	trigonal bipyramidal

	Geometrical Parameters (Å)					
	C=O - Me^{2+}					
complex	d(Thr-Me)	d(Pro-Me)	d(Val-Me)	d(*allo*-Ile-Me)	d(Tyr-Me)	d(ATA-Me)
$[cadA+Mg]^{2+}$	1,99	2,06	2,12	4,21	2,08	2,08
$[cadA+Ca]^{2+}$	2,32	2,45	2,42	2,47	2,43	2,47
$[cadA+Sr]^{2+}$	2,48	2,61	2,59	2,64	2,59	2,66
$[cadA+Zn]^{2+}$	1,96	2,06	2,13	4,32	2,07	2,04
$[cadA+Cu]^{2+}$	1,93	1,96	2,29	4,30	1,95	2,06

d: distance (A°)

	Geometrical Parameters (Å)			
	C=O - O=C			
complex	d(Pro-Tyr)	d(Thr-ATA)	d(ATA-Val)	d(Val-Thr)
$[cadA+Mg]^{2+}$	4,09	3,75	2,93	3,83
$[cadA+Ca]^{2+}$	4,86	4,41	3,07	4,44
$[cadA+Sr]^{2+}$	5,16	4,98	3,09	4,54
$[cadA+Zn]^{2+}$	4,04	3,64	2,95	3,82
$[cadA+Cu]^{2+}$	5,11	2,65	3,95	2,90

	Charge Distrubution (NBO)						
	Me	OThr	OPro	OVal	O*allo*Ile	OTyr	OATA
cadA	-	-0,633	-0,648	-0,632	-0,614	-0,68	-0,612
$[cadA+Mg]^{2+}$ ext	1,48	-0,794	-0,748	-0,761	-0,632	-0,76	-0,729
$[cadA+Ca]^{2+}$ ext	1,604	-0,779	-0,727	-0,754	-0,709	-0,74	-0,714
$[cadA+Sr]^{2+}$ ext *	1,595	-0,531	-0,457	-0,475	-0,408	-0,45	-0,465
$[cadA+Zn]^{2+}$ ext	1,369	-0,772	-0,73	-0,741	-0,632	-0,75	-0,717
$[cadA+Cu]^{2+}$ ext	1,228	-0,728	-0,711	-0,704	-0,631	-0,74	-0,692

* Mulliken charges

Table S7.2. ΔG differences between the two sets of low energy conformers for cadA metal complexes

Conformer	B3LYP	*ΔG°* (kcal/mol)	*ΔH°* (kcal/mol)
$[cadA+Mg]^{2+}$ int	6-31G(d)	8,85	11,03
$[cadA+Mg]^{2+}$	6-31G(d)	0,00	0,00
$[cadA+Ca]^{2+}$ int	6-31G(d)	11.55	13,56
$[cadA+Ca]^{2+}$	6-31G(d)	0,00	0,00
$[cadA+Sr]^{2+}$ int	LANL2DZ	17,11	21,36
$[cadA+Sr]^{2+}$ int	6-31G(d) / LANL2DZ	13,32	15,80
$[cadA+Sr]^{2+}$	LANL2DZ	0,00	0,00
$[cadA+Sr]^{2+}$	6-31G(d) / LANL2DZ	0,00	0,00
$[cadA+Zn]^{2+}$ int	6-31G(d)	9,51	10,91
$[cadA+Zn]^{2+}$	6-31G(d)	0,00	0,00

Part 8. Wortmannin

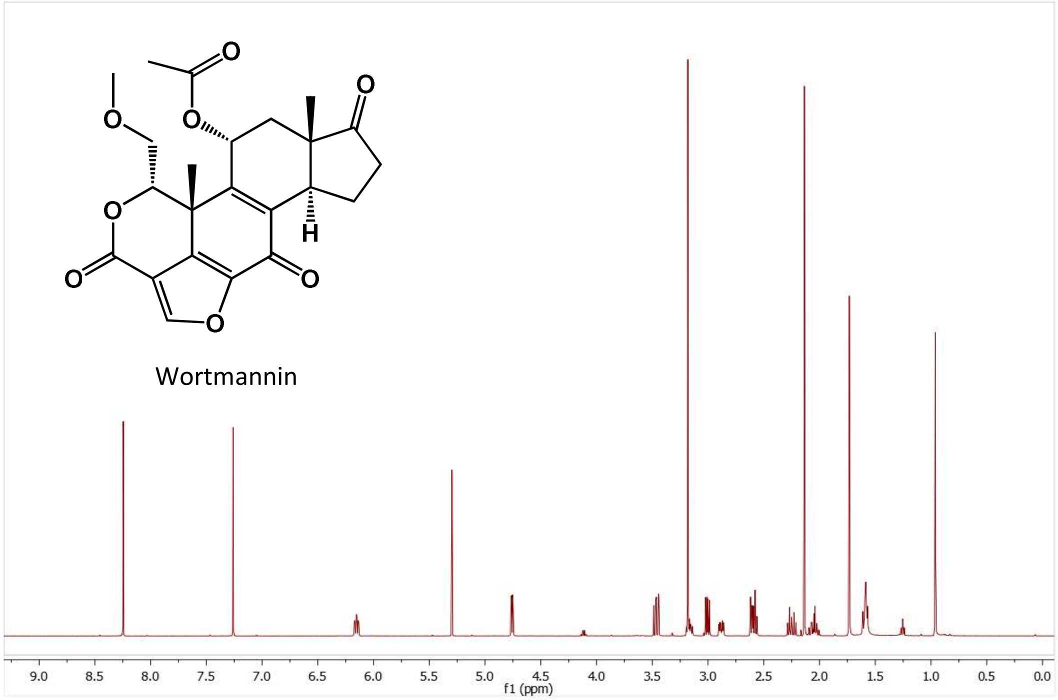

Figure S8.1. ^{1}H NMR of wortmannin

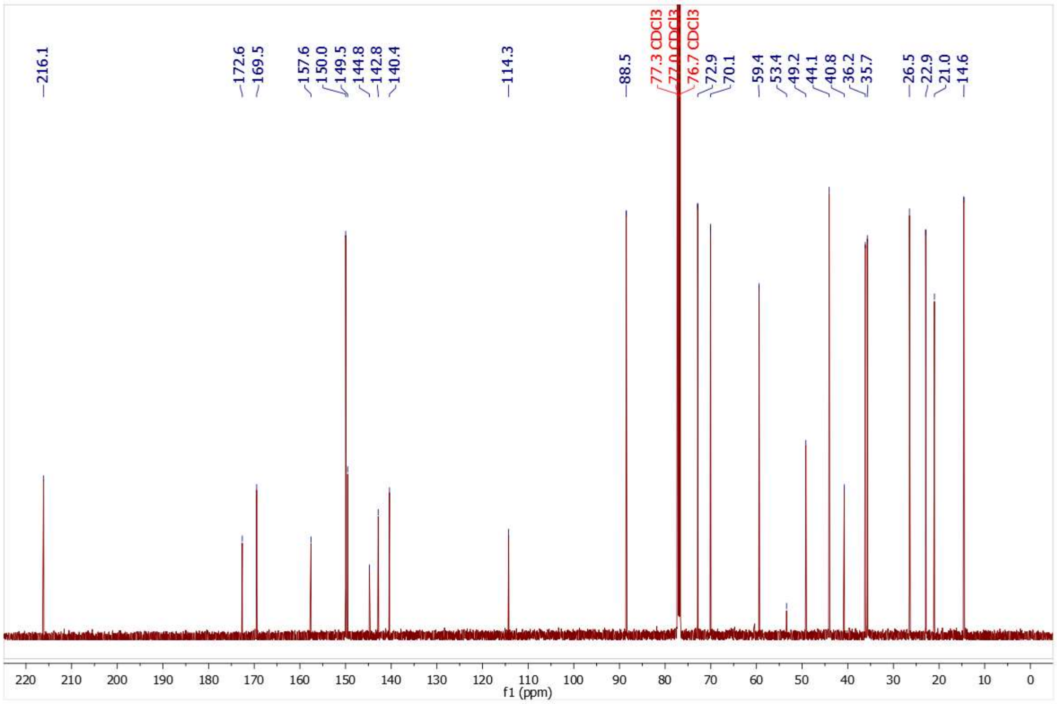

Figure S8.2. ^{13}C NMR of wortmannin

Part 9. **Isolation and cultivation of Antarctic fungal strains**

Samples (around 10 g) were aseptically taken from soil at 0–10 cm depth. After being collected, the samples were stored in sealed sterile bags or sterile flasks and transported to the station, where they were kept at 4°C until processed for incubation and isolation. Isolation was done on Potato Dextrose Agar (PDA) media. For the isolation protocol, a portion of the soil was resuspended in a minimal volume of saline solution supplemented with 1% tween 20 and then homogenized in a vortex mixer for 15 min. After that, 100 µL of the resulting homogenate was spread onto Petri plates with PDA. The plates were incubated at 15°C for 7-21 days under natural lighting conditions. After incubation, the fungi that grew were transferred to another PDA plate to obtain the pure culture of *Cadophora malorum,* that was maintained on PDA medium at 4°C in the Culture Collection in the Argentinean Antarctic Institute (IAA). Genomic DNA extraction was performed using a commercial kit (FastDNA™ Spin Kit, MP Biomedicals). The nuclear rDNA ITS1-5.8S-ITS2 regions (ITS) were amplified by PCR using the ITS4/ITS5 primer pair using the methods according to standard protocols, as described by Kurtzman [7]. The PCR products were purified and sequenced in MACROGEN (Korea). Sequences were analyzed and edited, when necessary, using DNA Dragon software [8]. *Cadophora malorum* identification was performed by comparison with the GenBank and UNITE data bases.

[7]. C. Kurtzman, J. W. Fell, T. Boekhout (Eds.) The yeasts: a taxonomic study., Elsevier (2011).

[8]. D. Hepperle. DNA Dragon 1.4. 1–DNA Sequence Contig Assembler Software. 2011 Available at: www. dna-dragon. com.

Part 10. **Antifungal assay**

The antifungal analysis of the peptides was carried out using the Kirby-Bauer disk-diffusion method [9]. The tested strains (*Candida albicans*, *C. haemulonni, Trichosporon* sp., *Rhyzopus* sp., *Aspergillus fumigatus* (susceptible (S) and resistant (R)) and *A. lentulus*) were cultured on PDA at 30°C for 36 hours. The fungal or yeast colonies were suspended in a sterile saline solution up to 0.5 MacFarland scale turbidity standard (10^7 spores ml^{-1} suspension). Each suspension (100 µl) was separately inoculated on PDA plates using a Drigalski spatula. Several Whatman® Antibiotic Assay Discs were placed in each plate. Two volumes (5 and 10 ml) with a 5 mmol/ml concentration of each peptide and a combination of both peptides were tested. Methanol and a 10 mg/ml methanolic solution of cycloheximide were used as negative and positive controls. Plates were incubated at 30°C for up to 2 days and the results were expressed as absence or presence of growth near the disks.

[9]. A. W. Bauer, W. M. M. Kirby, J. C. Sherris, M. Turck, *Am. J. Clin. Pathol*, 1966, **45**, 493-496.

APPENDIX Standard orientation

[cadA+Mg]$^{2+}$: $C_{39}H_{53}MgN_7O_9(2+)$

Center Number	Atomic Number	Atomic Type	Coordinates (Angstroms) X	Y	Z
1	6	0	6.977184	-0.811253	-0.541351
2	6	0	5.780671	-1.449886	-0.865648
3	6	0	4.614728	-0.705064	-1.116122
4	6	0	4.683753	0.711033	-1.026233
5	6	0	5.878363	1.343244	-0.718226
6	6	0	7.026862	0.580775	-0.470381
7	7	0	3.495366	1.471787	-1.329888
8	6	0	3.381679	-1.404992	-1.550727
9	6	0	2.405674	1.640156	-0.529418
10	6	0	2.497405	2.206921	0.895833
11	7	0	1.267609	1.980509	1.658427
12	6	0	-0.049499	2.244157	1.325542
13	6	0	-0.517509	3.518077	0.562732
14	7	0	-1.046255	3.128464	-0.740421
15	6	0	-2.378299	3.073443	-1.014523
16	6	0	-2.709854	2.590717	-2.451711
17	7	0	-3.088009	1.163850	-2.383199
18	6	0	-2.221650	0.167999	-2.358146
19	6	0	-2.749102	-1.281453	-2.292102
20	7	0	-2.422628	-1.835762	-0.969426
21	6	0	-1.207267	-2.304376	-0.692144
22	6	0	-0.943617	-2.864216	0.721887
23	7	0	0.485024	-3.206769	0.830492
24	6	0	1.490706	-2.383212	0.538713
25	6	0	2.935269	-2.930157	0.349893
26	7	0	3.163038	-2.687190	-1.099840
27	8	0	2.562405	-0.889987	-2.359264
28	8	0	1.278576	1.447595	-1.045594
29	6	0	3.619656	1.599718	1.796020
30	8	0	-0.924847	1.495098	1.757679
31	6	0	2.961404	1.463582	3.175735
32	6	0	1.521462	1.104877	2.818981
33	6	0	0.400746	4.764806	0.477070
34	6	0	-0.310831	5.893319	-0.292856
35	6	0	0.873755	5.256020	1.869543
36	6	0	-0.232037	5.721095	2.828053
37	8	0	-3.269850	3.362692	-0.232227
38	6	0	-3.860055	3.398197	-3.046101
39	8	0	-0.971758	0.365523	-2.397143
40	6	0	-4.241364	-1.475476	-2.607594
41	8	0	-4.939865	-0.735786	-1.598028
42	6	0	-4.661926	-2.941785	-2.644256
43	8	0	-0.291713	-2.289034	-1.558324
44	6	0	-1.432878	-1.965021	1.902317
45	6	0	-2.942097	-1.870176	2.029224
46	6	0	-3.593586	-0.627534	1.910279
47	6	0	-4.973996	-0.525416	2.056311
48	6	0	-5.737271	-1.667158	2.333652
49	6	0	-5.102574	-2.911786	2.464890
50	6	0	-3.721382	-3.002911	2.316770
51	8	0	-7.077582	-1.502240	2.452945
52	8	0	1.309357	-1.167259	0.271662
53	6	0	3.328514	-4.364259	0.773922

54	6	0	3.161391	-4.574909	2.289848
55	6	0	2.709674	-5.503688	-0.056199
56	1	0	7.872668	-1.398976	-0.367428
57	1	0	5.750941	-2.529414	-0.970641
58	1	0	5.911556	2.427098	-0.681371
59	1	0	7.961538	1.081482	-0.237635
60	1	0	3.181948	1.277115	-2.279418
61	1	0	2.683722	3.279107	0.748359
62	1	0	-1.407155	3.798425	1.135284
63	1	0	-0.392819	2.770036	-1.421987
64	1	0	-1.825339	2.645925	-3.091511
65	1	0	-4.060485	0.930645	-2.172684
66	1	0	-2.163656	-1.838200	-3.029165
67	1	0	-3.114536	-1.736310	-0.222440
68	1	0	-1.474424	-3.820059	0.786563
69	1	0	0.713092	-4.172230	1.024993
70	1	0	3.576680	-2.232732	0.895770
71	1	0	2.449639	-3.137906	-1.672406
72	1	0	4.517422	2.221058	1.797832
73	1	0	3.902574	0.608752	1.432257
74	1	0	3.451347	0.707101	3.794877
75	1	0	2.990619	2.414961	3.716636
76	1	0	1.420456	0.045434	2.553136
77	1	0	0.795239	1.328130	3.601527
78	1	0	1.297125	4.508209	-0.107963
79	1	0	0.296924	6.803859	-0.270933
80	1	0	-0.473780	5.626836	-1.340806
81	1	0	-1.286085	6.129449	0.145139
82	1	0	1.459074	4.470595	2.363826
83	1	0	1.566688	6.089837	1.699552
84	1	0	0.210535	6.075022	3.764672
85	1	0	-0.813619	6.548750	2.410991
86	1	0	-0.930707	4.916383	3.084565
87	1	0	-4.721501	3.384682	-2.372430
88	1	0	-4.145773	3.008689	-4.027799
89	1	0	-3.553156	4.440777	-3.167064
90	1	0	-4.418219	-1.026563	-3.596665
91	1	0	-5.891325	-0.925130	-1.650695
92	1	0	-5.723038	-3.017979	-2.905597
93	1	0	-4.100029	-3.492676	-3.405850
94	1	0	-4.514029	-3.426362	-1.674210
95	1	0	-1.007593	-2.419554	2.805772
96	1	0	-0.998900	-0.964869	1.812207
97	1	0	-3.010056	0.272097	1.729017
98	1	0	-5.473602	0.434749	1.979350
99	1	0	-5.685398	-3.800232	2.697339
100	1	0	-3.248617	-3.974403	2.453011
101	1	0	-7.497634	-2.329133	2.740146
102	1	0	4.406617	-4.394437	0.568723
103	1	0	3.617096	-5.524825	2.583477
104	1	0	3.653795	-3.782161	2.864439
105	1	0	2.113303	-4.611363	2.610667
106	1	0	3.181819	-6.449534	0.225080
107	1	0	2.878422	-5.370218	-1.130034
108	1	0	1.631835	-5.640350	0.099049
109	12	0	0.674116	-0.442257	-1.617085

[cadA+Cu]$^{2+}$: $C_{39}H_{53}CuN_7O_9(2+,2)$

Center Number	Atomic Number	Atomic Type	Coordinates (Angstroms) X	Y	Z
1	6	0	6.946934	-0.496533	-0.476231
2	6	0	5.794103	-1.210524	-0.803074
3	6	0	4.581573	-0.540898	-1.036401
4	6	0	4.558647	0.873873	-0.930401
5	6	0	5.707566	1.583917	-0.620039
6	6	0	6.904446	0.894987	-0.386280
7	7	0	3.314485	1.542109	-1.228125
8	6	0	3.379285	-1.298193	-1.460165
9	6	0	2.232837	1.661931	-0.418937
10	6	0	2.308467	2.260851	0.991457
11	7	0	1.082973	2.033897	1.757591
12	6	0	-0.235503	2.284643	1.422016
13	6	0	-0.705776	3.542288	0.633951
14	7	0	-1.200133	3.128278	-0.675979
15	6	0	-2.522516	3.111831	-0.997233
16	6	0	-2.815071	2.578955	-2.428479
17	7	0	-3.112708	1.132146	-2.339927
18	6	0	-2.196864	0.185480	-2.274045
19	6	0	-2.642475	-1.288613	-2.223415
20	7	0	-2.296376	-1.837575	-0.907465
21	6	0	-1.069241	-2.255480	-0.627652
22	6	0	-0.808926	-2.880344	0.752723
23	7	0	0.615472	-3.211020	0.869189
24	6	0	1.613786	-2.346268	0.643486
25	6	0	3.059328	-2.871557	0.414901
26	7	0	3.234233	-2.596811	-1.036210
27	8	0	2.524968	-0.807422	-2.245830
28	8	0	1.103808	1.406433	-0.917520
29	6	0	3.438983	1.671573	1.897007
30	8	0	-1.106775	1.540072	1.868565
31	6	0	2.780110	1.533828	3.276314
32	6	0	1.342817	1.163751	2.921315
33	6	0	0.197103	4.801328	0.554012
34	6	0	-0.522520	5.916245	-0.228044
35	6	0	0.649261	5.303602	1.949292
36	6	0	-0.473581	5.736411	2.903069
37	8	0	-3.429864	3.470961	-0.264833
38	6	0	-4.001732	3.306418	-3.051313
39	8	0	-0.959659	0.465151	-2.285585
40	6	0	-4.119737	-1.564089	-2.552830
41	8	0	-4.865235	-0.859500	-1.553452
42	6	0	-4.457709	-3.051783	-2.587374
43	8	0	-0.146127	-2.175978	-1.497607
44	6	0	-1.346187	-2.041108	1.958759
45	6	0	-2.859315	-2.009813	2.069060
46	6	0	-3.557948	-0.789398	1.990055
47	6	0	-4.942978	-0.746847	2.121261
48	6	0	-5.664239	-1.927115	2.344690
49	6	0	-4.982606	-3.150973	2.435196
50	6	0	-3.597493	-3.182744	2.301246
51	8	0	-7.010663	-1.818995	2.452841
52	8	0	1.411485	-1.128130	0.447323
53	6	0	3.487530	-4.305454	0.802782
54	6	0	3.333444	-4.547148	2.315000
55	6	0	2.887569	-5.443788	-0.042802

56	1	0	7.880763	-1.024836	-0.313651
57	1	0	5.834220	-2.288640	-0.919772
58	1	0	5.670237	2.667275	-0.572172
59	1	0	7.805437	1.452998	-0.151196
60	1	0	3.006900	1.305656	-2.169965
61	1	0	2.486294	3.331749	0.828663
62	1	0	-1.610591	3.818864	1.183579
63	1	0	-0.541008	2.705801	-1.314306
64	1	0	-1.926493	2.673080	-3.058138
65	1	0	-4.077890	0.847131	-2.162709
66	1	0	-2.021155	-1.804457	-2.961670
67	1	0	-3.001634	-1.796856	-0.164953
68	1	0	-1.332689	-3.842390	0.760908
69	1	0	0.854009	-4.183204	1.006928
70	1	0	3.705331	-2.173825	0.953650
71	1	0	2.516237	-3.059635	-1.593080
72	1	0	4.327457	2.305808	1.897056
73	1	0	3.733923	0.683113	1.536496
74	1	0	3.275535	0.781724	3.896393
75	1	0	2.803233	2.486546	3.815165
76	1	0	1.250430	0.104334	2.655453
77	1	0	0.615419	1.385521	3.703088
78	1	0	1.102881	4.556071	-0.021434
79	1	0	0.074950	6.833628	-0.209424
80	1	0	-0.675014	5.639433	-1.274928
81	1	0	-1.503383	6.144369	0.201354
82	1	0	1.253834	4.534125	2.445465
83	1	0	1.319768	6.156327	1.783505
84	1	0	-0.045110	6.110666	3.838353
85	1	0	-1.082544	6.541205	2.480305
86	1	0	-1.144718	4.909708	3.163356
87	1	0	-4.868599	3.259715	-2.386285
88	1	0	-4.252956	2.882195	-4.028055
89	1	0	-3.752536	4.361885	-3.189643
90	1	0	-4.309065	-1.129553	-3.545914
91	1	0	-5.804234	-1.102926	-1.609589
92	1	0	-5.508839	-3.186854	-2.864330
93	1	0	-3.855255	-3.575187	-3.337261
94	1	0	-4.299209	-3.522498	-1.612094
95	1	0	-0.911668	-2.511099	2.849504
96	1	0	-0.950838	-1.022722	1.905875
97	1	0	-3.007403	0.138278	1.852611
98	1	0	-5.479397	0.195361	2.076344
99	1	0	-5.532610	-4.069761	2.625746
100	1	0	-3.088547	-4.139720	2.406220
101	1	0	-7.401873	-2.670613	2.706871
102	1	0	4.564301	-4.309782	0.588101
103	1	0	3.798128	-5.498733	2.588605
104	1	0	3.822864	-3.761157	2.901234
105	1	0	2.286567	-4.595907	2.637965
106	1	0	3.397967	-6.379542	0.203358
107	1	0	3.025814	-5.278321	-1.116608
108	1	0	1.819361	-5.623878	0.135200
109	29	0	0.621518	-0.383373	-1.568006

[cadA+Zn]$^{2+}$: $C_{39}H_{53}N_7O_9Zn(2+)$

Center Number	Atomic Number	Atomic Type	Coordinates (Angstroms) X	Y	Z
1	6	0	6.936033	-0.681454	-0.450074
2	6	0	5.757086	-1.349782	-0.779126
3	6	0	4.573727	-0.632494	-1.026521
4	6	0	4.604266	0.783756	-0.927272
5	6	0	5.780672	1.445611	-0.612969
6	6	0	6.948397	0.711028	-0.369169
7	7	0	3.393441	1.508925	-1.227549
8	6	0	3.354471	-1.352663	-1.458356
9	6	0	2.302624	1.648939	-0.423476
10	6	0	2.386223	2.235394	0.992292
11	7	0	1.152594	2.021099	1.750647
12	6	0	-0.161902	2.282930	1.405977
13	6	0	-0.619278	3.539916	0.608367
14	7	0	-1.086131	3.131154	-0.713257
15	6	0	-2.402335	3.072689	-1.052928
16	6	0	-2.658358	2.552934	-2.493851
17	7	0	-3.023027	1.123449	-2.403445
18	6	0	-2.153728	0.132915	-2.341797
19	6	0	-2.676700	-1.312762	-2.234215
20	7	0	-2.370676	-1.812247	-0.885473
21	6	0	-1.167559	-2.291705	-0.583222
22	6	0	-0.926408	-2.827884	0.842180
23	7	0	0.502305	-3.153408	0.979398
24	6	0	1.497631	-2.326947	0.661382
25	6	0	2.943685	-2.863805	0.459741
26	7	0	3.140940	-2.625715	-0.994931
27	8	0	2.539386	-0.852376	-2.286750
28	8	0	1.177547	1.426926	-0.932575
29	6	0	3.503264	1.634275	1.903612
30	8	0	-1.042185	1.548936	1.853159
31	6	0	2.837997	1.513432	3.281412
32	6	0	1.397297	1.159142	2.923061
33	6	0	0.286137	4.797748	0.549826
34	6	0	-0.420557	5.919340	-0.234371
35	6	0	0.720182	5.289350	1.954532
36	6	0	-0.415490	5.702122	2.902033
37	8	0	-3.333090	3.380787	-0.325703
38	6	0	-3.788279	3.330086	-3.162426
39	8	0	-0.902096	0.344143	-2.381660
40	6	0	-4.163921	-1.524478	-2.564327
41	8	0	-4.880073	-0.750194	-1.593933
42	6	0	-4.576668	-2.993389	-2.548481
43	8	0	-0.247775	-2.320888	-1.446926
44	6	0	-1.452907	-1.921057	1.999584
45	6	0	-2.966287	-1.855894	2.096381
46	6	0	-3.639616	-0.627291	1.955419
47	6	0	-5.024705	-0.551762	2.072323
48	6	0	-5.770813	-1.706363	2.342685
49	6	0	-5.114196	-2.937250	2.495574
50	6	0	-3.728834	-3.001935	2.375938
51	8	0	-7.116139	-1.567415	2.433758
52	8	0	1.300598	-1.116804	0.377017
53	6	0	3.354596	-4.291785	0.886082
54	6	0	3.211383	-4.492671	2.405791

55	6	0	2.730906	-5.441034	0.073410
56	1	0	7.846838	-1.246029	-0.279129
57	1	0	5.755200	-2.429274	-0.889772
58	1	0	5.785743	2.529676	-0.568162
59	1	0	7.869394	1.234262	-0.131488
60	1	0	3.085732	1.307676	-2.177428
61	1	0	2.576321	3.305239	0.833390
62	1	0	-1.535278	3.813777	1.140351
63	1	0	-0.402850	2.749180	-1.351395
64	1	0	-1.744760	2.602467	-3.091201
65	1	0	-3.997508	0.887845	-2.202716
66	1	0	-2.078410	-1.896744	-2.939058
67	1	0	-3.073676	-1.686073	-0.152300
68	1	0	-1.448338	-3.788918	0.906140
69	1	0	0.738345	-4.114571	1.185626
70	1	0	3.588491	-2.157389	0.989365
71	1	0	2.425691	-3.089498	-1.554759
72	1	0	4.402238	2.253759	1.904186
73	1	0	3.785306	0.639559	1.548675
74	1	0	3.321432	0.760230	3.909623
75	1	0	2.870447	2.469599	3.813624
76	1	0	1.291181	0.097176	2.669739
77	1	0	0.669649	1.396140	3.700207
78	1	0	1.199665	4.554014	-0.014023
79	1	0	0.181785	6.833300	-0.207494
80	1	0	-0.566681	5.647414	-1.283428
81	1	0	-1.403544	6.151680	0.188051
82	1	0	1.326934	4.519901	2.448088
83	1	0	1.384953	6.149298	1.804103
84	1	0	0.000781	6.075513	3.843158
85	1	0	-1.031089	6.502187	2.479784
86	1	0	-1.078409	4.865428	3.151446
87	1	0	-4.680000	3.324419	-2.529290
88	1	0	-4.023659	2.911489	-4.145450
89	1	0	-3.486948	4.372665	-3.296765
90	1	0	-4.328250	-1.115141	-3.572450
91	1	0	-5.830034	-0.945448	-1.652428
92	1	0	-5.633628	-3.085338	-2.821232
93	1	0	-4.001666	-3.571342	-3.279625
94	1	0	-4.440278	-3.437258	-1.557488
95	1	0	-1.035282	-2.352242	2.917840
96	1	0	-1.038334	-0.913520	1.900652
97	1	0	-3.070066	0.282453	1.780651
98	1	0	-5.541536	0.397768	1.978729
99	1	0	-5.683863	-3.835575	2.722592
100	1	0	-3.240122	-3.963002	2.528936
101	1	0	-7.525702	-2.400337	2.718711
102	1	0	4.429736	-4.315757	0.664967
103	1	0	3.677739	-5.437436	2.699218
104	1	0	3.706689	-3.692588	2.967513
105	1	0	2.168326	-4.533866	2.742079
106	1	0	3.212865	-6.381823	0.354951
107	1	0	2.883267	-5.315014	-1.003820
108	1	0	1.656287	-5.582476	0.245348
109	30	0	0.678505	-0.468280	-1.556527

[cadA+Ca]$^{2+}$: $C_{39}H_{53}CaN_7O_9(2+)$

Center Number	Atomic Number	Atomic Type	Coordinates (Angstroms) X	Y	Z
1	6	0	7.007344	-1.088071	0.021414
2	6	0	5.795736	-1.618725	-0.416253
3	6	0	4.734236	-0.784640	-0.808970
4	6	0	4.937319	0.622273	-0.764638
5	6	0	6.158161	1.146441	-0.355942
6	6	0	7.188667	0.294458	0.052206
7	7	0	3.905781	1.492249	-1.274825
8	6	0	3.494368	-1.390854	-1.371337
9	6	0	2.813237	2.036480	-0.682201
10	6	0	2.751351	2.721329	0.711510
11	7	0	1.465137	2.356824	1.345572
12	6	0	0.267304	2.299394	0.655207
13	6	0	-0.488546	3.576679	0.215187
14	7	0	-1.352625	3.236955	-0.905442
15	6	0	-2.612001	2.745594	-0.723114
16	6	0	-3.381912	2.381457	-2.014044
17	7	0	-3.630324	0.928911	-2.015531
18	6	0	-2.664490	0.041414	-2.222854
19	6	0	-3.003178	-1.468561	-2.159316
20	7	0	-2.568659	-2.018692	-0.865287
21	6	0	-1.302932	-2.398459	-0.633173
22	6	0	-0.969586	-2.989686	0.761094
23	7	0	0.442053	-3.409864	0.775486
24	6	0	1.482609	-2.591150	0.570004
25	6	0	2.910923	-3.152503	0.313565
26	7	0	3.195102	-2.705498	-1.072765
27	8	0	2.764206	-0.774104	-2.181544
28	8	0	1.757029	2.072446	-1.358794
29	6	0	3.814572	2.372738	1.791436
30	8	0	-0.326088	1.209194	0.554278
31	6	0	3.003787	1.937463	3.028208
32	6	0	1.730476	1.362600	2.411565
33	6	0	0.286742	4.911620	0.015710
34	6	0	-0.546320	5.930358	-0.785435
35	6	0	0.747454	5.532051	1.363131
36	6	0	-0.367761	6.073294	2.268706
37	8	0	-3.131323	2.595948	0.374716
38	6	0	-4.712730	3.130971	-2.072175
39	8	0	-1.481531	0.388848	-2.463026
40	6	0	-4.476208	-1.825947	-2.421189
41	8	0	-5.203743	-1.253637	-1.323667
42	6	0	-4.717920	-3.326969	-2.548167
43	8	0	-0.414457	-2.277377	-1.503721
44	6	0	-1.316881	-2.063214	1.971837
45	6	0	-2.795290	-1.804296	2.180382
46	6	0	-3.332394	-0.520729	1.972783
47	6	0	-4.682562	-0.260664	2.179627
48	6	0	-5.533173	-1.287026	2.611995
49	6	0	-5.014732	-2.571469	2.832139
50	6	0	-3.660296	-2.819306	2.618795
51	8	0	-6.842058	-0.975453	2.788129
52	8	0	1.348186	-1.353258	0.475360
53	6	0	3.247773	-4.648282	0.521652
54	6	0	3.049506	-5.079315	1.985938

55	6	0	2.599753	-5.623525	-0.477785
56	1	0	7.815990	-1.752228	0.308813
57	1	0	5.678370	-2.693639	-0.502685
58	1	0	6.301956	2.221823	-0.370599
59	1	0	8.138878	0.714952	0.366313
60	1	0	3.643745	1.209618	-2.218535
61	1	0	2.777794	3.790263	0.470425
62	1	0	-1.183341	3.712158	1.058264
63	1	0	-0.970373	3.283864	-1.839941
64	1	0	-2.778917	2.601754	-2.900437
65	1	0	-4.529234	0.573462	-1.688303
66	1	0	-2.384520	-1.932337	-2.931643
67	1	0	-3.242526	-2.000193	-0.100528
68	1	0	-1.550575	-3.911286	0.868601
69	1	0	0.626863	-4.402564	0.809098
70	1	0	3.556427	-2.570256	0.977403
71	1	0	2.482852	-3.035158	-1.722926
72	1	0	4.464868	3.227719	1.990331
73	1	0	4.449036	1.550751	1.465207
74	1	0	3.541561	1.209946	3.641872
75	1	0	2.759624	2.798175	3.658005
76	1	0	1.888464	0.354316	2.010951
77	1	0	0.878308	1.334662	3.093994
78	1	0	1.181924	4.698235	-0.583726
79	1	0	-0.027730	6.894611	-0.801437
80	1	0	-0.690474	5.623534	-1.825523
81	1	0	-1.535493	6.089340	-0.345103
82	1	0	1.331689	4.799438	1.930371
83	1	0	1.432464	6.354669	1.121480
84	1	0	0.063608	6.465618	3.195100
85	1	0	-0.920461	6.890676	1.796523
86	1	0	-1.092100	5.300953	2.553832
87	1	0	-5.295824	2.939039	-1.166640
88	1	0	-5.288702	2.832005	-2.953194
89	1	0	-4.532557	4.207936	-2.133430
90	1	0	-4.764058	-1.331852	-3.361028
91	1	0	-6.133548	-1.530245	-1.371325
92	1	0	-5.779142	-3.522052	-2.738208
93	1	0	-4.155311	-3.743386	-3.390353
94	1	0	-4.430646	-3.856548	-1.634808
95	1	0	-0.892917	-2.565949	2.849548
96	1	0	-0.784131	-1.117219	1.854700
97	1	0	-2.688948	0.295784	1.654223
98	1	0	-5.084134	0.734711	2.021736
99	1	0	-5.664880	-3.369969	3.182775
100	1	0	-3.275006	-3.817360	2.820564
101	1	0	-7.317826	-1.725922	3.179342
102	1	0	4.327233	-4.691637	0.324923
103	1	0	3.468809	-6.077935	2.139249
104	1	0	3.557392	-4.398501	2.678620
105	1	0	1.995686	-5.125581	2.285036
106	1	0	3.023889	-6.621852	-0.334869
107	1	0	2.797750	-5.338092	-1.516394
108	1	0	1.513628	-5.728928	-0.361457
109	20	0	0.534154	-0.044786	-1.395176

[cadA+Sr]$^{2+}$ $C_{39}H_{53}N_7O_9Sr(2+)$

Center Number	Atomic Number	Atomic Type	Coordinates (Angstroms) X	Y	Z
1	6	0	6.964951	-1.086022	0.708468
2	6	0	5.832170	-1.618687	0.072668
3	6	0	4.838096	-0.774428	-0.477005
4	6	0	5.020061	0.643166	-0.380674
5	6	0	6.173240	1.168031	0.213621
6	6	0	7.138524	0.308361	0.773955
7	7	0	4.053312	1.499574	-1.037157
8	6	0	3.714489	-1.383030	-1.251941
9	6	0	2.943308	2.159894	-0.590400
10	6	0	2.713417	2.867732	0.783582
11	7	0	1.431199	2.336792	1.325717
12	6	0	0.235770	2.332979	0.613356
13	6	0	-0.550627	3.631445	0.265409
14	7	0	-1.487299	3.316591	-0.820941
15	6	0	-2.707027	2.735906	-0.572751
16	6	0	-3.573773	2.402930	-1.813254
17	7	0	-3.821239	0.940572	-1.836557
18	6	0	-2.880885	0.057276	-2.198433
19	6	0	-3.198702	-1.462155	-2.126621
20	7	0	-2.704915	-2.009963	-0.840424
21	6	0	-1.417266	-2.394387	-0.667091
22	6	0	-1.016053	-2.960225	0.720493
23	7	0	0.391993	-3.430328	0.685330
24	6	0	1.460758	-2.642734	0.440699
25	6	0	2.906347	-3.232156	0.288400
26	7	0	3.354803	-2.703158	-1.030798
27	8	0	3.113995	-0.744551	-2.192160
28	8	0	1.953244	2.229097	-1.410067
29	6	0	3.682341	2.649934	1.957214
30	8	0	-0.339110	1.219095	0.381447
31	6	0	3.228359	1.315049	2.598038
32	6	0	1.708814	1.221797	2.303927
33	6	0	0.228504	4.971514	0.053693
34	6	0	-0.613609	6.010738	-0.727816
35	6	0	0.697726	5.572735	1.417840
36	6	0	-0.424935	6.141978	2.314952
37	8	0	-3.121082	2.475924	0.584628
38	6	0	-4.913579	3.156220	-1.753500
39	8	0	-1.708819	0.420231	-2.576097
40	6	0	-4.686091	-1.819654	-2.328758
41	8	0	-5.372098	-1.205283	-1.173409
42	6	0	-4.952542	-3.326061	-2.416563
43	8	0	-0.559127	-2.278711	-1.606660
44	6	0	-1.274986	-1.955985	1.905231
45	6	0	-2.744396	-1.769672	2.259502
46	6	0	-3.373575	-0.512116	2.089400
47	6	0	-4.711925	-0.318016	2.460533
48	6	0	-5.446465	-1.384933	3.014065
49	6	0	-4.837723	-2.645531	3.199897
50	6	0	-3.495466	-2.825347	2.826191
51	8	0	-6.771620	-1.122756	3.344947
52	8	0	1.347293	-1.384909	0.256270
53	6	0	3.159309	-4.762119	0.404782
54	6	0	2.840597	-5.306776	1.820614

55	6	0	2.519686	-5.619420	-0.716033
56	1	0	7.723211	-1.749197	1.113096
57	1	0	5.740560	-2.693419	-0.051711
58	1	0	6.338357	2.240796	0.201872
59	1	0	8.032728	0.726895	1.226325
60	1	0	3.920143	1.215467	-2.011932
61	1	0	2.638974	3.927705	0.524681
62	1	0	-1.181747	3.753233	1.162027
63	1	0	-1.214280	3.518885	-1.774929
64	1	0	-3.030806	2.658236	-2.730885
65	1	0	-4.688747	0.566297	-1.436153
66	1	0	-2.610929	-1.918586	-2.928784
67	1	0	-3.350387	-1.980751	-0.049271
68	1	0	-1.624289	-3.853993	0.906532
69	1	0	0.548424	-4.423194	0.809337
70	1	0	3.493841	-2.733862	1.068126
71	1	0	2.825775	-3.085131	-1.815895
72	1	0	3.533714	3.476292	2.660932
73	1	0	4.732134	2.656639	1.667169
74	1	0	3.757959	0.469380	2.145364
75	1	0	3.434725	1.295783	3.671425
76	1	0	1.431257	0.253369	1.882648
77	1	0	1.101006	1.405067	3.195840
78	1	0	1.116632	4.762151	-0.559989
79	1	0	-0.101522	6.980412	-0.713071
80	1	0	-0.745307	5.736476	-1.781679
81	1	0	-1.607698	6.149939	-0.289179
82	1	0	1.245882	4.815931	1.994091
83	1	0	1.408800	6.381454	1.195878
84	1	0	-0.002177	6.494845	3.263189
85	1	0	-0.930138	6.993712	1.847334
86	1	0	-1.188871	5.392224	2.560865
87	1	0	-5.446574	2.923938	-0.824780
88	1	0	-5.543328	2.894299	-2.611125
89	1	0	-4.741758	4.237523	-1.778722
90	1	0	-5.031257	-1.328307	-3.250207
91	1	0	-6.292008	-1.523611	-1.067399
92	1	0	-6.021822	-3.517683	-2.570187
93	1	0	-4.420596	-3.764661	-3.269503
94	1	0	-4.637249	-3.844508	-1.504524
95	1	0	-0.734404	-2.374921	2.764583
96	1	0	-0.817491	-0.992793	1.664182
97	1	0	-2.825352	0.331584	1.676744
98	1	0	-5.188806	0.649219	2.342806
99	1	0	-5.395722	-3.467329	3.644897
100	1	0	-3.029518	-3.793209	3.009279
101	1	0	-7.206298	-1.851368	3.834576
102	1	0	4.247693	-4.853208	0.277114
103	1	0	3.295342	-6.295742	1.942441
104	1	0	3.246933	-4.659077	2.608441
105	1	0	1.767119	-5.433405	2.016312
106	1	0	2.799000	-6.669023	-0.573015
107	1	0	2.880294	-5.332821	-1.711837
108	1	0	1.422300	-5.577826	-0.732806
109	38	0	0.603653	0.026443	-1.782685

----

cadA#1 $C_{39}H_{53}N_7O_9$

Center Number	Atomic Number	Atomic Type	Coordinates (Angstroms) X	Y	Z
1	6	0	2.665244	-4.670921	-2.732908
2	6	0	2.780165	-4.398372	-1.374146
3	6	0	1.717849	-3.849245	-0.637028
4	6	0	0.467133	-3.651602	-1.288346
5	6	0	0.373625	-3.887734	-2.670714
6	6	0	1.462632	-4.384145	-3.380895
7	7	0	-0.659697	-3.290732	-0.525817
8	6	0	1.878281	-3.543419	0.818941
9	6	0	-1.886250	-2.869654	-0.971974
10	6	0	-3.004879	-2.967305	0.084872
11	7	0	-3.996110	-1.901990	-0.098017
12	6	0	-3.683445	-0.643864	0.292480
13	6	0	-4.726676	0.481584	0.094455
14	7	0	-4.028502	1.761080	0.059754
15	6	0	-3.413676	2.193794	-1.081755
16	6	0	-2.269224	3.208023	-0.828673
17	7	0	-1.158307	2.457871	-0.230295
18	6	0	-1.076620	2.213751	1.092898
19	6	0	-0.051294	1.132631	1.495159
20	7	0	1.326368	1.505066	1.184145
21	6	0	1.881648	1.276042	-0.023033
22	6	0	3.419493	1.284538	-0.079342
23	7	0	3.829524	-0.120254	-0.163743
24	6	0	3.984794	-0.904493	0.934757
25	6	0	4.197400	-2.407844	0.661048
26	7	0	3.089677	-3.129391	1.307499
27	8	0	0.933430	-3.694221	1.615874
28	8	0	-2.129898	-2.521283	-2.123905
29	6	0	-3.837167	-4.251749	-0.136585
30	8	0	-2.583045	-0.397079	0.804564
31	6	0	-4.896014	-3.819204	-1.163307
32	6	0	-5.231599	-2.376219	-0.754054
33	6	0	-5.830537	0.466198	1.189054
34	6	0	-5.259528	0.610306	2.610064
35	6	0	-6.914722	1.532202	0.927621
36	6	0	-7.651086	1.403841	-0.410622
37	8	0	-3.700079	1.786981	-2.205325
38	6	0	-1.832049	3.919499	-2.102488
39	8	0	-1.807254	2.768742	1.932746
40	6	0	-0.194449	0.793187	2.990869
41	8	0	0.159621	1.931993	3.780573
42	6	0	0.665560	-0.382166	3.435650
43	8	0	1.216799	0.975828	-1.025563
44	6	0	3.954944	2.073648	-1.286544
45	6	0	3.643090	3.554321	-1.213315
46	6	0	2.548561	4.102657	-1.898108
47	6	0	2.253306	5.462529	-1.825310
48	6	0	3.056504	6.308309	-1.051976
49	6	0	4.153284	5.781526	-0.359923
50	6	0	4.436441	4.418620	-0.446698
51	8	0	2.725715	7.634779	-1.015346
52	8	0	3.963435	-0.470881	2.087936
53	6	0	5.563832	-2.863893	1.232544
54	6	0	5.794903	-4.369011	1.047146

55	6	0	6.713691	-2.054542	0.612448
56	1	0	3.501637	-5.099636	-3.275737
57	1	0	3.702834	-4.644415	-0.862049
58	1	0	-0.564845	-3.694759	-3.170154
59	1	0	1.356910	-4.572495	-4.445593
60	1	0	-0.526965	-3.442524	0.474298
61	1	0	-2.583421	-2.912521	1.091386
62	1	0	-5.192367	0.354299	-0.884303
63	1	0	-3.583114	2.045761	0.927800
64	1	0	-2.596511	3.932442	-0.077796
65	1	0	-0.555806	1.897533	-0.835668
66	1	0	-0.300618	0.247558	0.902714
67	1	0	1.908082	1.784071	1.964836
68	1	0	3.835968	1.681499	0.848730
69	1	0	3.713671	-0.568674	-1.064435
70	1	0	4.163744	-2.600804	-0.410809
71	1	0	3.030656	-2.948101	2.306530
72	1	0	-4.307253	-4.535215	0.811284
73	1	0	-3.225989	-5.093396	-0.473618
74	1	0	-4.469245	-3.830484	-2.169612
75	1	0	-5.781058	-4.460719	-1.155740
76	1	0	-5.487067	-1.749099	-1.612876
77	1	0	-6.069949	-2.352171	-0.048669
78	1	0	-6.313783	-0.517758	1.124925
79	1	0	-6.063281	0.505581	3.346821
80	1	0	-4.502393	-0.149585	2.828505
81	1	0	-4.802986	1.595079	2.768276
82	1	0	-7.644585	1.462453	1.744974
83	1	0	-6.461947	2.529041	1.004220
84	1	0	-8.475364	2.123802	-0.465722
85	1	0	-6.992424	1.599193	-1.264292
86	1	0	-8.077507	0.400861	-0.537426
87	1	0	-1.513925	3.200799	-2.863544
88	1	0	-1.003762	4.600061	-1.885479
89	1	0	-2.663398	4.498331	-2.515241
90	1	0	-1.251293	0.542231	3.143568
91	1	0	-0.509670	2.602616	3.543784
92	1	0	0.493443	-0.571962	4.500126
93	1	0	0.407102	-1.287885	2.876665
94	1	0	1.732472	-0.187617	3.288958
95	1	0	5.037962	1.912647	-1.329490
96	1	0	3.522363	1.644692	-2.197113
97	1	0	1.916429	3.454530	-2.499263
98	1	0	1.410174	5.882077	-2.366174
99	1	0	4.785127	6.435285	0.237385
100	1	0	5.297937	4.025552	0.088635
101	1	0	3.358012	8.107189	-0.449423
102	1	0	5.534694	-2.644873	2.308820
103	1	0	6.718050	-4.671001	1.553931
104	1	0	4.973217	-4.960927	1.461729
105	1	0	5.905146	-4.626025	-0.014101
106	1	0	7.671489	-2.384842	1.028170
107	1	0	6.618672	-0.981946	0.811285
108	1	0	6.755300	-2.197136	-0.474718

----

cadA#2 $C_{39}H_{53}N_7O_9$

Center Number	Atomic Number	Atomic Type	Coordinates (Angstroms) X	Y	Z
1	6	0	-1.085719	5.580849	-2.526718
2	6	0	-1.268016	5.198750	-1.201849
3	6	0	-0.368645	4.335464	-0.554622
4	6	0	0.790817	3.903820	-1.255206
5	6	0	0.946212	4.261299	-2.604865
6	6	0	0.014486	5.085382	-3.229607
7	7	0	1.782426	3.184294	-0.564783
8	6	0	-0.602027	3.916553	0.862081
9	6	0	2.806951	2.437318	-1.086306
10	6	0	3.912280	2.109670	-0.066122
11	7	0	4.461402	0.764586	-0.265716
12	6	0	3.729321	-0.306121	0.132741
13	6	0	4.374889	-1.710227	0.049737
14	7	0	3.331341	-2.722118	0.086020
15	6	0	2.590084	-3.018833	-1.018567
16	6	0	1.264274	-3.751324	-0.691766
17	7	0	0.343360	-2.779640	-0.091452
18	6	0	0.389495	-2.438932	1.214411
19	6	0	-0.496256	-1.232202	1.591865
20	7	0	-1.891259	-1.351000	1.158308
21	6	0	-2.309504	-0.930537	-0.054717
22	6	0	-3.761409	-0.413708	-0.119268
23	7	0	-3.667707	1.051499	-0.192887
24	6	0	-3.299597	1.823715	0.856511
25	6	0	-3.072445	3.317237	0.554397
26	7	0	-1.885039	3.734439	1.307766
27	8	0	0.341448	3.762433	1.657921
28	8	0	2.909643	2.121083	-2.268367
29	6	0	5.139541	3.021199	-0.302082
30	8	0	2.585200	-0.157371	0.579725
31	6	0	5.970202	2.243592	-1.335184
32	6	0	5.790473	0.778594	-0.910106
33	6	0	5.412845	-1.943785	1.185577
34	6	0	4.811133	-1.758210	2.589285
35	6	0	6.099414	-3.320475	1.064674
36	6	0	6.851692	-3.563614	-0.248382
37	8	0	2.916655	-2.713177	-2.164342
38	6	0	0.642290	-4.392043	-1.926482
39	8	0	1.123654	-2.999777	2.040406
40	6	0	-0.462829	-0.946825	3.131547
41	8	0	-1.735321	-0.519205	3.622433
42	6	0	0.638283	0.050255	3.480790
43	8	0	-1.572285	-0.862263	-1.047577
44	6	0	-4.522287	-0.952641	-1.341979
45	6	0	-4.744097	-2.449913	-1.281220
46	6	0	-3.852733	-3.342560	-1.890414
47	6	0	-4.046810	-4.722817	-1.821335
48	6	0	-5.149857	-5.235891	-1.130293
49	6	0	-6.052231	-4.359850	-0.514514
50	6	0	-5.844324	-2.985478	-0.594348
51	8	0	-5.401738	-6.575870	-1.026347
52	8	0	-3.153492	1.380422	2.002329
53	6	0	-4.309103	4.199933	0.896564
54	6	0	-5.511545	3.850748	0.007256

55	6	0	-4.688597	4.175322	2.384470
56	1	0	-1.792041	6.253572	-3.002692
57	1	0	-2.110006	5.595397	-0.644273
58	1	0	1.809114	3.899463	-3.145963
59	1	0	0.166129	5.360228	-4.269629
60	1	0	1.702737	3.264542	0.448445
61	1	0	3.521549	2.196308	0.951097
62	1	0	4.875341	-1.804345	-0.916284
63	1	0	2.856538	-2.858874	0.976257
64	1	0	1.463911	-4.508682	0.072135
65	1	0	-0.196293	-2.168662	-0.709552
66	1	0	-0.052459	-0.384355	1.053112
67	1	0	-2.538160	-1.256454	1.937011
68	1	0	-4.298389	-0.666938	0.798373
69	1	0	-3.635205	1.454499	-1.121384
70	1	0	-2.857482	3.434302	-0.508050
71	1	0	-1.890255	3.403450	2.268885
72	1	0	5.689555	3.123812	0.639652
73	1	0	4.858713	4.023026	-0.638197
74	1	0	5.559209	2.394877	-2.336719
75	1	0	7.022636	2.539309	-1.342941
76	1	0	5.821424	0.089228	-1.758684
77	1	0	6.567980	0.481957	-0.197010
78	1	0	6.189754	-1.179428	1.058428
79	1	0	5.594100	-1.870730	3.347065
80	1	0	4.364098	-0.766817	2.715510
81	1	0	4.038090	-2.504648	2.808387
82	1	0	6.805298	-3.407281	1.901248
83	1	0	5.350756	-4.109490	1.211185
84	1	0	7.400871	-4.510952	-0.206204
85	1	0	6.174256	-3.619521	-1.107769
86	1	0	7.579586	-2.766512	-0.445096
87	1	0	0.460754	-3.644712	-2.704854
88	1	0	-0.305269	-4.872023	-1.664441
89	1	0	1.315934	-5.148199	-2.339897
90	1	0	-0.274098	-1.894430	3.640403
91	1	0	-1.970271	0.335928	3.210488
92	1	0	0.707347	0.170408	4.566702
93	1	0	1.603156	-0.291779	3.095771
94	1	0	0.428086	1.031016	3.034016
95	1	0	-5.482938	-0.427917	-1.393697
96	1	0	-3.953782	-0.691881	-2.241761
97	1	0	-2.990334	-2.955802	-2.426511
98	1	0	-3.345690	-5.398506	-2.306514
99	1	0	-6.908942	-4.767125	0.013925
100	1	0	-6.557160	-2.315521	-0.118700
101	1	0	-4.708715	-7.069403	-1.494685
102	1	0	-3.994836	5.223344	0.650494
103	1	0	-6.323485	4.564918	0.180427
104	1	0	-5.253339	3.891490	-1.057670
105	1	0	-5.899994	2.849121	0.224382
106	1	0	-5.514997	4.871323	2.564486
107	1	0	-3.854146	4.484388	3.023010
108	1	0	-5.010308	3.178184	2.702303

----

cadA#3 $C_{39}H_{53}N_7O_9$

Center Number	Atomic Number	Atomic Type	Coordinates (Angstroms) X	Y	Z
1	6	0	-2.134310	4.939641	-1.778607
2	6	0	-2.131303	4.374703	-0.507573
3	6	0	-1.007689	3.697092	-0.006272
4	6	0	0.174099	3.653883	-0.795677
5	6	0	0.149879	4.192115	-2.093007
6	6	0	-0.993988	4.821259	-2.575921
7	7	0	1.353776	3.130142	-0.237304
8	6	0	-1.037546	3.074966	1.353772
9	6	0	2.492158	2.723499	-0.884073
10	6	0	3.704568	2.559107	0.050389
11	7	0	4.551539	1.428567	-0.343224
12	6	0	4.128432	0.168321	-0.070533
13	6	0	5.094922	-1.007986	-0.350192
14	7	0	4.340317	-2.249254	-0.404537
15	6	0	3.625446	-2.600235	-1.510743
16	6	0	2.547340	-3.676944	-1.229440
17	7	0	1.455473	-3.044203	-0.480531
18	6	0	1.501359	-2.851180	0.855158
19	6	0	0.376781	-1.952339	1.412346
20	7	0	-0.974721	-2.357813	1.014363
21	6	0	-1.553934	-1.910275	-0.121692
22	6	0	-3.088008	-1.761059	-0.076550
23	7	0	-3.355044	-0.321259	0.038655
24	6	0	-3.130319	0.385166	1.170695
25	6	0	-3.299733	1.912085	1.059735
26	7	0	-2.204138	2.526094	1.817667
27	8	0	-0.035267	3.075600	2.090324
28	8	0	2.593618	2.582716	-2.099700
29	6	0	4.655244	3.770358	-0.097084
30	8	0	3.010222	-0.029997	0.420486
31	6	0	5.583618	3.356772	-1.249716
32	6	0	5.796977	1.854170	-1.014024
33	6	0	6.227915	-1.094628	0.713420
34	6	0	5.687819	-1.231508	2.147485
35	6	0	7.231653	-2.224380	0.399996
36	6	0	7.934814	-2.116890	-0.957588
37	8	0	3.791132	-2.097503	-2.620964
38	6	0	2.024189	-4.315430	-2.510279
39	8	0	2.402508	-3.299242	1.578490
40	6	0	0.440160	-1.848772	2.973124
41	8	0	-0.862775	-1.787577	3.557015
42	6	0	1.309467	-0.672659	3.408531
43	8	0	-0.915292	-1.555118	-1.120913
44	6	0	-3.752054	-2.344808	-1.336115
45	6	0	-5.257878	-2.187391	-1.364922
46	6	0	-5.876563	-1.280117	-2.236557
47	6	0	-7.261278	-1.119831	-2.258809
48	6	0	-8.061610	-1.876701	-1.396562
49	6	0	-7.464715	-2.793410	-0.522168
50	6	0	-6.078637	-2.941907	-0.514710
51	8	0	-9.414014	-1.683155	-1.456071
52	8	0	-2.809882	-0.141152	2.243981
53	6	0	-4.689974	2.405243	1.558452
54	6	0	-5.826731	1.861445	0.680622

55	6	0	-4.947243	2.118900	3.045373
56	1	0	-3.013435	5.463116	-2.140747
57	1	0	-3.007379	4.480625	0.123713
58	1	0	1.038591	4.123375	-2.704363
59	1	0	-0.982021	5.243227	-3.576903
60	1	0	1.322838	3.071904	0.780135
61	1	0	3.367349	2.420208	1.080946
62	1	0	5.540111	-0.862569	-1.336759
63	1	0	3.972623	-2.599985	0.477547
64	1	0	2.979037	-4.437836	-0.572452
65	1	0	0.744670	-2.520912	-0.997761
66	1	0	0.569609	-0.964384	0.972961
67	1	0	-1.576985	-2.504006	1.820603
68	1	0	-3.490373	-2.241918	0.818730
69	1	0	-3.493615	0.188093	-0.825026
70	1	0	-3.192082	2.202509	0.014375
71	1	0	-2.065849	2.098889	2.729648
72	1	0	5.221462	3.890872	0.832876
73	1	0	4.117801	4.703434	-0.286785
74	1	0	5.083567	3.516575	-2.208498
75	1	0	6.528296	3.906894	-1.253143
76	1	0	5.949633	1.300530	-1.944870
77	1	0	6.663070	1.681955	-0.365083
78	1	0	6.778124	-0.147082	0.659734
79	1	0	6.519464	-1.221903	2.860354
80	1	0	5.012304	-0.411210	2.411103
81	1	0	5.144870	-2.172868	2.295386
82	1	0	7.988653	-2.216107	1.195297
83	1	0	6.719953	-3.192532	0.473747
84	1	0	8.709798	-2.886095	-1.050469
85	1	0	7.240378	-2.253815	-1.793980
86	1	0	8.420313	-1.140527	-1.079640
87	1	0	1.601040	-3.560089	-3.179362
88	1	0	1.253629	-5.055119	-2.274351
89	1	0	2.838339	-4.815424	-3.042842
90	1	0	0.868706	-2.779569	3.350885
91	1	0	-1.314869	-0.973001	3.259830
92	1	0	1.416581	-0.665982	4.497980
93	1	0	2.300455	-0.739073	2.950854
94	1	0	0.858835	0.278549	3.096279
95	1	0	-3.298551	-1.872851	-2.214739
96	1	0	-3.477300	-3.405910	-1.377116
97	1	0	-5.268238	-0.691786	-2.919868
98	1	0	-7.732281	-0.417040	-2.939404
99	1	0	-8.082100	-3.391407	0.144359
100	1	0	-5.632823	-3.665348	0.164239
101	1	0	-9.850755	-2.264931	-0.812584
102	1	0	-4.658569	3.495956	1.431540
103	1	0	-6.778287	2.312091	0.981875
104	1	0	-5.668459	2.094854	-0.378905
105	1	0	-5.929880	0.774612	0.775648
106	1	0	-5.905326	2.558414	3.343233
107	1	0	-4.172992	2.556532	3.684671
108	1	0	-4.990289	1.043920	3.248592
